# State-Space Analysis of Time-Varying Higher-Order Spike Correlation for Multiple Neural Spike Train Data

**DOI:** 10.1371/journal.pcbi.1002385

**Published:** 2012-03-08

**Authors:** Hideaki Shimazaki, Shun-ichi Amari, Emery N. Brown, Sonja Grün

**Affiliations:** 1 RIKEN Brain Science Institute, Wako-shi, Saitama, Japan; 2 Department of Brain and Cognitive Sciences, Massachusetts Institute of Technology, Cambridge, Massachusetts, United States of America; 3 Department of Anesthesia and Critical Care, Massachusetts General Hospital, Boston, Massachusetts, United States of America; 4 Division of Health Sciences and Technology, Harvard Medical School/Massachusetts Institute of Technology, Cambridge, Massachusetts, United States of America; 5 Institute of Neuroscience and Medicine (INM-6), Computational and Systems Neuroscience, Research Center Jülich, Jülich, Germany; 6 Theoretical Systems Neurobiology, RWTH Aachen University, Aachen, Germany; Indiana University, United States of America

## Abstract

Precise spike coordination between the spiking activities of multiple neurons is suggested as an indication of coordinated network activity in active cell assemblies. Spike correlation analysis aims to identify such cooperative network activity by detecting excess spike synchrony in simultaneously recorded multiple neural spike sequences. Cooperative activity is expected to organize dynamically during behavior and cognition; therefore currently available analysis techniques must be extended to enable the estimation of multiple time-varying spike interactions between neurons simultaneously. In particular, new methods must take advantage of the simultaneous observations of multiple neurons by addressing their higher-order dependencies, which cannot be revealed by pairwise analyses alone. In this paper, we develop a method for estimating time-varying spike interactions by means of a state-space analysis. Discretized parallel spike sequences are modeled as multi-variate binary processes using a log-linear model that provides a well-defined measure of higher-order spike correlation in an information geometry framework. We construct a recursive Bayesian filter/smoother for the extraction of spike interaction parameters. This method can simultaneously estimate the dynamic pairwise spike interactions of multiple single neurons, thereby extending the Ising/spin-glass model analysis of multiple neural spike train data to a nonstationary analysis. Furthermore, the method can estimate dynamic higher-order spike interactions. To validate the inclusion of the higher-order terms in the model, we construct an approximation method to assess the goodness-of-fit to spike data. In addition, we formulate a test method for the presence of higher-order spike correlation even in nonstationary spike data, e.g., data from awake behaving animals. The utility of the proposed methods is tested using simulated spike data with known underlying correlation dynamics. Finally, we apply the methods to neural spike data simultaneously recorded from the motor cortex of an awake monkey and demonstrate that the higher-order spike correlation organizes dynamically in relation to a behavioral demand.

## Author Summary

Nearly half a century ago, the Canadian psychologist D. O. Hebb postulated the formation of assemblies of tightly connected cells in cortical recurrent networks because of changes in synaptic weight (Hebb's learning rule) by repetitive sensory stimulation of the network. Consequently, the activation of such an assembly for processing sensory or behavioral information is likely to be expressed by precisely coordinated spiking activities of the participating neurons. However, the available analysis techniques for multiple parallel neural spike data do not allow us to reveal the detailed structure of transiently active assemblies as indicated by their dynamical pairwise and higher-order spike correlations. Here, we construct a state-space model of dynamic spike interactions, and present a recursive Bayesian method that makes it possible to trace multiple neurons exhibiting such precisely coordinated spiking activities in a time-varying manner. We also formulate a hypothesis test of the underlying dynamic spike correlation, which enables us to detect the assemblies activated in association with behavioral events. Therefore, the proposed method can serve as a useful tool to test Hebb's cell assembly hypothesis.

## Introduction

Precise spike coordination within the spiking activities of multiple single neurons is discussed as an indication of coordinated network activity in the form of cell assemblies [1] comprising neuronal information processing. Possible theoretical mechanisms and conditions for generating and maintaining such precise spike coordination have been proposed on the basis of neuronal network models [2]–[4]. The effect of synchronous spiking activities on downstream neurons has been theoretically investigated and it was demonstrated that these are more effective in generating output spikes [5]. Assembly activity was hypothesized to organize dynamically as a result of sensory input and/or in relation to behavioral context [6]–[10]. Supportive experimental evidence was provided by findings of the presence of excess spike synchrony occurring dynamically in relation to stimuli [11]–[14], behavior [14]–[19], or internal states such as memory retention, expectation, and attention [8], [20]–[23].

Over the years, various statistical tools have been developed to analyze the dependency between neurons, with continuous improvement in their applicability to neuronal experimental data (see [24]–[26] for recent reviews). The cross-correlogram [27] was the first analysis method for detecting the correlation between pairs of neurons and focused on the detection of stationary correlation. The joint-peri stimulus time histogram (JPSTH) introduced by [11], [28] is an extension of the cross-correlogram that allows a time resolved analysis of the correlation dynamics between a pair of neurons. This method relates the joint spiking activity of two neurons to a trigger event, as was done in the peri-stimulus time histogram (PSTH) [29]–[31] for estimating the time dependent firing rate of a single neuron. The Unitary Event analysis method [25], [32], [33] further extended the correlation analysis to enable it to test the statistical dependencies between multiple, nonstationary spike sequences against a null hypothesis of full independence among neurons. Staude et al. developed a test method (CuBIC) that enables the detection of higher-order spike correlation by computing the cumulants of the bin-wise population spike counts [34], [35].

In the last decade, other model-based methods have been developed that make it possible to capture the dependency among spike sequences by direct statistical modeling of the parallel spike sequences. Two related approaches based on a generalized linear framework are being extensively investigated. One models the spiking activities of single neurons as a continuous-time point process or as a discrete-time Bernoulli process. The point process intensities (instantaneous spike rates) or Bernoulli success probabilities of individual neurons are modeled in a generalized linear manner using a log link function or a logit link function, respectively [36]–[38]. The dependency among neurons is modeled by introducing coupling terms that incorporate the spike history of other observed neurons into the instantaneous spike rate [38]–[40]. Recent development in causality analysis for point process data [41] makes it possible to perform formal statistical significance tests of the causal interactions in these models. Typically, the models additionally include the covariate stimulus signals in order to investigate receptive field properties of neurons, i.e., the relations between neural spiking activities and the known covariate signals. However, they are not suitable for capturing instantaneous, synchronous spiking activities, which are likely to be induced by an unobserved external stimulus or a common input from an unobserved set of neurons. Recently, a model was proposed to dissociate instantaneous synchrony from the spike-history dependencies; it additionally includes a common, non-spike driven latent signal [42], [43]. These models provide a concise description of the multiple neural spike train data by assuming independent spiking activities across neurons conditional on these explanatory variables. As a result, however, they do not aim to directly model the joint distribution of instantaneous spiking activities of multiple neurons.

In contrast, an alternative approach, which we will follow and extend in this paper, directly models the instantaneous, joint spiking activities by treating the neuronal system as an ensemble binary pattern generator. In this approach, parallel spike sequences are represented as binary events occurring in discretized time bins, and are modeled as a multivariate Bernoulli distribution using a multinomial logit link function. The dependencies among the binary units are modeled in the generalized linear framework by introducing undirected pairwise and higher-order interaction terms for instantaneous, synchronous spike events. This statistical model is referred to as the ‘log-linear model’ [44], [45], or Ising/spin-glass model if the model contains only lower-order interactions. The latter is also referred to as the maximum entropy model when these parameters are estimated under the maximum likelihood principle. In contrast to the former, biologically-inspired network-style modeling, the latter approach using a log-linear model was motivated by the computational theory of artificial neural networks originating from the stationary distribution of a Boltzmann machine [46], [47], which is in turn the stochastic analogue of the Hopfield network model [48], [49] for associative memory.

The merit of the log-linear model is its ability to provide a well-defined measure of spike correlation. While the cross-correlogram and JPSTH provide a measure of the marginal correlation of two neurons, these methods cannot distinguish direct pairwise correlations from correlations that are indirectly induced through other neurons. In contrast, a simultaneous pairwise analysis based on the log-linear model (an analogue of the Ising/spin-glass analysis in statistical mechanics) can sort out all of the pair-dependencies of the observed neurons. A further merit of the log-linear model is that it can provide a measure of the ‘pure’ higher-order spike correlation, i.e., a state that can not be explained by lower-order interactions. Using the viewpoint of an information geometry framework [50], Amari et al. [44], [45], [51] demonstrated that the higher-order spike correlations can be extracted from the higher-order parameters of the log-linear model (a.k.a. the natural or canonical parameters). The strengths of these parameters are interpreted in relation to the lower-order parameters of the dual orthogonal coordinates (a.k.a. the expectation parameters). The information contained in the higher-order spike interactions of a particular log-linear model can be extracted by measuring the distance (e.g., the Kullback-Leibler divergence) between the higher-order model and its projection to a lower-order model space, i.e., a manifold spanned by the natural parameters whose higher-order interaction terms are fixed at zero [44], [52]–[54].

Recently, a log-linear model that considered only up to pairwise interactions (i.e., an Ising/spin-glass model) was proposed as a model for parallel spike sequences. Its adequateness was shown by the fact that the firing rates and pairwise interactions explained more than 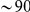
% of the data [55]–[57]. However, Roudi et al. [54] demonstrated that the small contribution of higher-order correlations found from their measure based on the Kullback-Leibler divergence could be an artifact caused by the small number of neurons analyzed. Other studies have reported that higher-order correlations are required to account for the dependencies between parallel spike sequences [58], [59], or for stimulus encoding [53], [60]. In [60], they reported the existence of triple-wise spike correlations in the spiking activity of the neurons in the visual cortex and showed their stimulus dependent changes. It should be noted, though, that these analyses assumed stationarity, both of the firing rates of individual neurons and of their spike correlations. This was possible because the authors restricted themselves to data recorded either from *in vitro* slices or from anesthetized animals. However, in order to assess the behavioral relevance of pairwise and higher-order spike correlations in awake behaving animals, it is necessary to appropriately correct for time-varying firing rates within an experimental trial and provide an algorithm that reliably estimates the time-varying spike correlations within multiple neurons.

We consider the presence of excess spike synchrony, in particular the excess synchrony explained by higher-order correlation, as an indicator of an active cell assembly. If some of the observed neurons are a subset of the neurons that comprise an assembly, they are likely to exhibit nearly completely synchronous spikes every time the assembly is activated. It may be that such spike patterns are not explained by mere pairwise correlations, but require higher-order correlations for explanation of their occurrence. One of the potential physiological mechanisms for higher-order correlated activity is a common input from a set of unobserved neurons to the assembly that includes the neurons under observation [25], [61]–[63]. Such higher-order activity is transient in nature and expresses a momentary snapshot of the neuronal dynamics. Thus, methods that are capable of evaluating time-varying, higher-order spike correlations are crucial to test the hypothesis that biological neuronal networks organize in dynamic cell assemblies for information processing. However, many of the current approaches based on the log-linear model [44], [45], [53], [55], [56], [61], [62], [64], [65] are not designed to capture their dynamics. Very recently two approaches were proposed for testing the presence of non-zero pairwise [66] and higher-order [67] correlations using a time-dependent formulation of a log-linear model. In contrast to these methods, the present paper aims to directly provide optimized estimates of the individual time-varying interactions with confidence intervals. This enables to identify short lasting, time-varying higher-order correlation and thus to relate them to behaviorally relevant time periods.

In this paper, we propose an approach to estimate the dynamic assembly activities from multiple neural spike train data using a ‘state-space log-linear’ framework. A state-space model offers a general framework for modeling time-dependent systems by representing its parameters (states) as a Markov process. Brown et al. [37] developed a recursive filtering algorithm for a point process observation model that is applicable to neural spike train data. Further, Smith and Brown [68] developed a paradigm for joint state-space and parameter estimation for point process observations using an expectation-maximization (EM) algorithm. Since then, the algorithm has been continuously improved and was successfully applied to experimental neuronal spike data from various systems [38], [69]–[71] (see [72] for a review). Here, we extend this framework, and construct a multivariate state-space model of multiple neural spike sequences by using the log-linear model to follow the dynamics of the higher-order spike interactions. Note that we assume for this analysis typical electrophysiological experiments in which multiple neural spike train data are repeatedly collected under identical experimental conditions (‘trials’). Thus, with the proposed method, we deal with the within-trial nonstationarity of the spike data that is expected in the recordings from awake behaving animals. We assume, however, that dynamics of the spiking statistics within trials, such as time-varying spike rates and higher-order interactions, are identical across the multiple experimental trials (across-trial stationarity).

To validate the necessity of including higher-order interactions in the model, we provide a method for evaluating the goodness-of-fit of the state-space model to the observed parallel spike sequences using the Akaike information criterion [73]. We then formulate a hypothesis test for the presence of the latent, time-varying spike interaction parameters by combining the Bayesian model comparison method [74]–[76] with a surrogate method. The latter test method provides us with a tool to detect assemblies that are momentarily activated, e.g., in association with behavioral events. We test the utility of these methods by applying them to simulated parallel spike sequences with known dependencies. Finally, we apply the methods to spike data of three neurons simultaneously recorded from motor cortex of an awake monkey and demonstrate that a triple-wise spike correlation dynamically organizes in relation to a behavioral demand.

The preliminary results were presented in the proceedings of the IEEE ICASSP meeting in 2009 [77], as well as in conference abstracts (Shimazaki et al., Neuro08, SAND4, NIPS08WS, Cosyne09, and CNS09).

## Results

### General formulation

#### Log-linear model of multiple neural spike sequences

We consider an ensemble spike pattern of 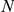 neurons. The state of each neuron is represented by a binary random variable, 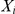 (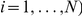 where ‘
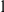
’ denotes a spike occurrence and ‘
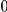
’ denotes silence. An ensemble spike pattern of 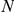 neurons is represented by a vector, 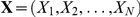, with the total number of possible spike patterns equal to 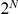. Let 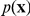, where 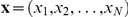 and 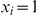 or 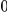 (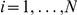), represent the joint probability mass function of the 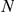-tuple binary random variables, 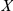. Because of the constraint 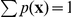, the probabilities of all the spike patterns are specified using 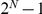 parameters. In information geometry [44],[50], these 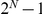 parameters are viewed as ‘coordinates’ that span a manifold composed of the set of all probability distributions, 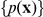. In the following, we consider two coordinate systems.

The logarithm of the probability mass function can be expanded as
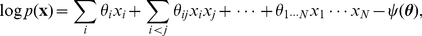
(1)where 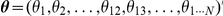. In this study, a prime indicates the transposition operation to a vector or matrix. 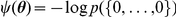 is a log normalization parameter to satisfy 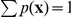. The log normalization parameter, 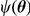, is a function of 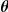. Eq. 1 is referred to as the log-linear model. The parameters 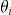, 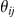,…, 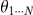 are the natural parameters of an exponential family distribution and form the ‘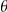-coordinates’. The 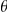-coordinates play a central role in this paper.

The other coordinates, called 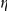-coordinates, can be constructed using the following expectation parameters:
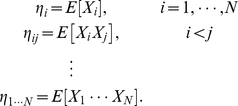
(2)These parameters represent the expected rates of joint spike occurrences among the neurons indexed by the subscripts. We denote the set of expectation parameters as a vector 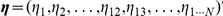.

Eqs. 1 and 2 can be compactly written using the following ‘multi-indices’ notation. Let 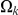 be collections of all the 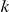-element subsets of a set with 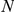 elements (i.e., a k-subset of 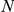 elements): 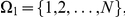 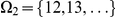, 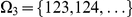, etc. We use the ‘multi-indices’ 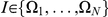 to represent the natural parameters and expectation parameters as 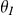 and 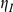, respectively. Similarly, let us denote the interaction terms in Eq. 1 as
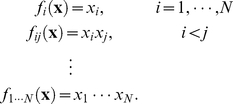
(3)Using 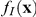 (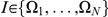), Eqs. 1 and 2 can now be compactly written as 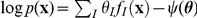 and 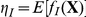, respectively.

The higher-order natural parameters in the log-linear model represent the strength of the higher-order spike interactions. Amari et al. [44], [45], [50], [51] proved that the 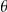
- and 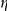-coordinates are dually ‘orthogonal’ coordinates and demonstrated that the natural parameters that are greater than or equal to the 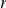
th-order, 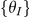 (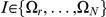, 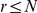), represent an excess or paucity of higher-order synchronous spikes in the 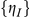 (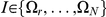) coordinates. To understand the potential influence of the higher-order natural parameters on the higher-order joint spike event rates, let us consider a log-linear model in which the parameters higher than or equal to the 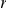
th-order vanish: 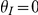 (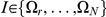). In the dual representation, the higher-order joint spike event rates, 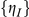 (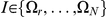), are chance coincidences expected from the lower-order joint spike event rates, 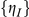 (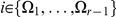). From this, it follows that the non-zero higher-order natural parameters that are greater than or equal to the 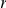
th-order of a full log-linear model represent the excess or paucity of the higher-order joint spike event rates, 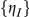 (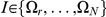), in comparison to their chance rates expected from the lower-order joint spike event rates, 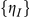 (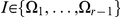).

However, in this framework, the excess or scarce synchronous spike events of 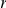 subset neurons reflected in 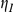 (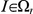) are *caused* not only by the non-zero 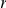
th-order interactions 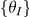 (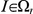), but also by all non-zero higher-order interactions 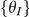 (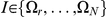). Therefore, one cannot extract the influence of pure 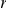
th-order spike interactions (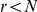) on the higher-order synchrony rates unless the parameters higher than the 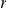
th-orders vanish, 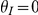 (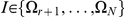). To formally extract the 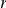
th-order spike interactions, let 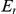 denote a log-linear model whose parameters higher than the 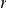
th-order are fixed at zero. By successively adding higher-order terms into the model, we can consider a set of log-linear models forming a hierarchical structure as 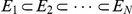. Here, 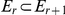 denotes that 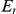 is a sub-manifold of 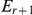 in the 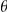-coordinates. In 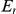, the set of non-zero 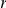
th-order natural parameters explains the excess or paucity of 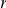
th-order synchronous spike events, 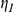 (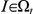), in comparison to their chance occurrence rates expected from the lower-order marginal rates, 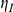 (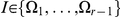). In a full model 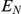, the last parameter in the log-linear model, 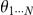, represents the pure 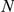
th-order spike correlation among the observed 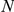 neurons. The information geometry theory developed by Amari and others [44], [45], [50], [51] provides a framework for illustrating the duality and orthogonal relations between the 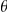
- and 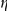-coordinates and singling out the higher-order correlations from these coordinates using hierarchical models. In the Methods subsection ‘Mathematical properties of log-linear model’ we describe the known properties of the log-linear model utilized in this study, and give references [44], [45], [50], [51] for further details on the information geometry approach to spike correlations.

#### State-space log-linear model of multiple neural spike sequences

To model the dynamics of the spike interactions, we extend the log-linear model to a time-dependent formulation. To do so, we parameterize the natural parameters as 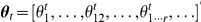 in discrete time steps 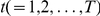. We denote the dimension of 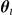 for an 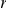
th-order model as 
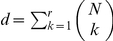
. The time-dependent log-linear model up to the 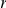-th order interactions (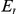) at time 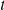 is then defined as
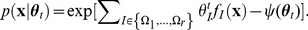
(4)The corresponding time-varying expectation parameters at time 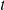 are written as 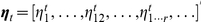, where each element (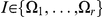) is given as
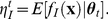
(5)


In neurophysiological experiments, neuronal responses are repeatedly obtained under identical experimental conditions (‘trials’). Thus, we here consider the parallel spike sequences repeatedly recorded simultaneously from 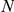 neurons over 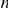 trials and align them to a trigger event. We model these simultaneous spike sequences as time-varying, multivariate binary processes by assuming that these parallel spike sequences are discretized in time into 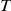 bins of bin-width 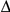. Let 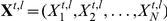 be the observed 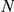-tuple binary variables in the 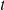-th bin of the 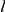-th trial, whereby a bin containing ‘
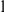
’ values indicates that one or more spikes occurred in the bin whereas ‘
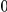
’ values indicate that no spike occurred in the bin. We regard the observed spike pattern 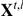 at time 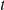 as a sample (from a total of 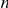 trials) from a joint probability mass function. An efficient estimator of 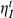 is the observed spike synchrony rate defined for the 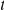-th bin as
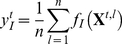
(6)for 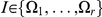. The synchrony rates up to the 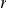-th order, 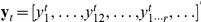, constitute a sufficient statistic for the log-linear model up to the 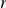-th order interaction.

Using Eqs. 4 and 6, the likelihood of the observed parallel spike sequences is given as
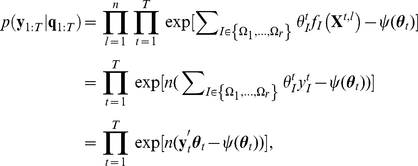
(7)with 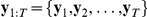 and 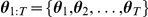. Here, we assumed that the observed spike patterns are conditionally independent across bins given the time-dependent natural parameters, and that the samples across trials are independent.

Our prior assumption about the time-dependent natural parameters is expressed by the following state equation
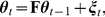
(8)for 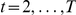. Matrix 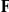 (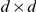 matrix) contains the first order autoregressive parameters. 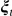 (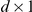 matrix) is a random vector drawn from a zero-mean multivariate normal distribution with covariance matrix 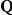 (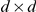 matrix). The initial values obey 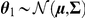, with 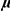 (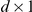 matrix) being the mean and 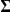 (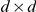 matrix) the covariance matrix. The parameters 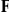, 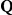, 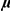, and 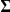 are called hyper-parameters. In the following, we denote the set of hyper-parameters by 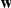: 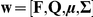.

Given the likelihood (Eq. 7) and prior distribution (Eq. 8), we aim at obtaining the posterior density
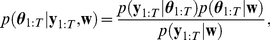
(9)using Bayes' theorem. The posterior density provides us with the most likely paths of the log-liner parameters given the spike data (maximum a posteriori, MAP, estimates) as well as the uncertainty in its estimation. The posterior density depends on the choice of the hyper-parameters, 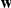. Here, the hyper-parameters, 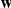, except for the covariance matrix 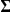 for the initial parameters, are optimized using the principle of maximizing the logarithm of the marginal likelihood (the denominator of Eq. 9, also referred to as the evidence),
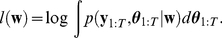
(10)


**Figure 1:**
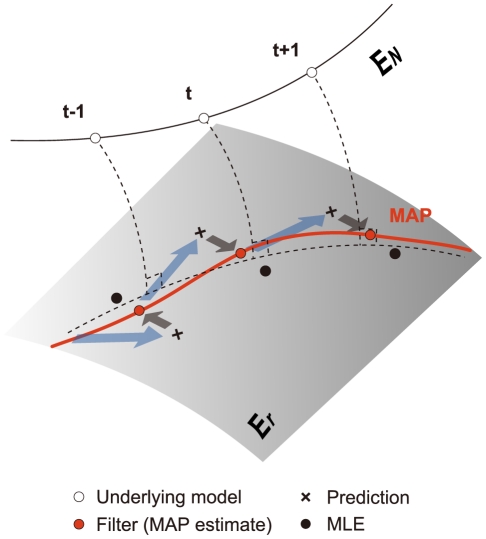
Geometric view of recursive filtering in subspace 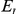. Each point in this figure represents a probability distribution, 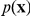, of an 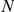-tuple binary variable, 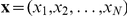. The underlying time-dependent model is represented by white circles in the space of 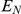. The dashed lines indicate projections of the underlying models to the model subspace, 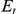. The maximum a posteriori (MAP) estimates of the underlying models projected on subspace 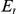 were obtained recursively: Starting from the MAP estimate at time 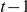 (filter estimate, red circle), the model at time 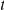 is predicted based on the prior knowledge of the state transition, Eq. 8 (blue arrow, prediction; black cross, a predicted distribution). The maximum likelihood estimate (MLE, black circle) for the spike data at time 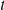 derived by Eq. 4 is expected to appear near the projection point of the underlying model at time 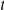 in 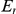. The filter distribution at time 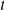 is obtained by correcting the prediction by the observation of data at time 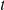 (black arrow). The filter estimation at time 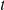 is used for predicting the model at time 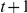 and so on. This recursive procedure allows us to retain past information while tracking the underlying time-dependent model based on the current observation.

For non-Gaussian observation models such as Eq. 7, the exact calculation of Eq. 10 is difficult (but see the approximate formula in the Methods section). Instead, we use the EM algorithm [68], [78] to efficiently combine the construction of the posterior density and the optimization of the hyper-parameters under the maximum likelihood principle. Using this algorithm, we iteratively construct a posterior density with the given hyper-parameters (E-step), and then use it to optimize the hyper-parameters (M-step). To obtain the posterior density (Eq. 9) in the E-step, we develop a nonlinear recursive Bayesian filter/smoother. The filter distribution is sequentially constructed by combining the prediction distribution for time 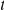 based on the state equation (Eq. 8) and the likelihood function for the spike data at time 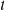, Eq. 4. Figure 1 illustrates the recursive filtering process in model subspace 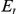. In combination with a fixed-interval smoothing algorithm, we derive the smooth posterior density (Eq. 9). The time-dependent log-linear parameters (natural parameters) are estimated as MAP estimates of the optimized smooth posterior density. In the Methods section, please refer to the subsection on ‘Bayesian estimation of dynamic spike interactions’ for the derivation of the optimization method for hyper-parameters, along with the filtering/smoothing methods. We summarize a method for estimating the dynamic spike interactions in Table 1.

**Table 1:** Method for estimating dynamic spike interactions.

**I**	**Preprocessing parallel spike data**		
	(1)	Align parallel spike sequences from 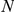 neurons at the onset of external clock that repeated 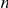 times.	
	(2)	Construct binary sequences 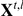 ( 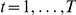 and 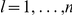 ) using 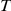 bins of width 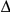 from the spike timing data.	
	(3)	Select 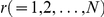 , the order of interactions included in the model. At each bin, compute the joint spike event rates up to the 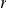 th order 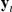 , using Eq. 6.	
**II**	**Optimized estimation of time-varying log-linear parameters**		
	(1)	Initialize the hyper parameters: 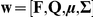 .†	
	(2)	E-step: Apply the recursive Bayesian filter/smoother to obtain posterior densities.	
		(i)	Filtering: For 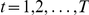 , recursively obtain
			the one-step prediction density, 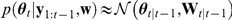 , using Eqs. 25 and 26,
			the filter density, 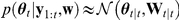 , using Eqs. 31 and 32.
		(ii)	Smoothing: For 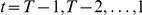 , recursively obtain
			the smooth density, 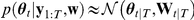 , using Eqs. 34 and 35.
	(3)	M-step: Optimize the hyper-parameters.	
			Update the hyper-parameters, 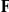 and 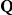 , using Eqs. 38 and 39, and 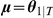 .
	(4)	Repeat (2) and (3) until the iterations satisfy a predetermined convergence criterion.‡	

**†:** In this study, we initialized the hyper-parameters using 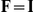, 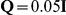, 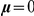 and used a fixed diagonal covariance matrix for an initial density, as 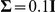, unless specified otherwise in the main text or figure captions.

**‡:** In our algorithm, we computed the approximate log marginal likelihood, 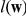, of the model using Eq. 45. We stopped the EM algorithm if the increment of the log marginal likelihood was smaller than 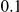.

### Application of state-space log-linear model to simulated spike data

#### Estimation of time-varying pairwise spike interaction

To demonstrate the utility of the developed methods for the analysis of dynamic spike correlations, we first consider a nonstationary pairwise spike correlation analysis. For this goal, we apply the state-space method to two examples of simulated spike data, with 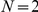 neurons. The dynamic spike correlation between two neurons can be analyzed by conceptually simpler histogram-based methods, e.g., a joint peri-stimulus time histogram (JPSTH). However, even for the pair-analysis, the proposed method can be advantageous in the following two aspects. First, the proposed method provides a credible interval (a Bayesian analogue of a confidence interval). Using the recursive Bayesian filtering/smoothing algorithm developed in the Methods section, we obtain the joint posterior density of the log-linear parameters (Eq. 9). The posterior density provides, not only the most likely path of the log-liner parameters (MAP estimates), but also the uncertainty in its estimation. The credible interval allows us to examine whether the pairwise spike correlation is statistically significant (but see the later section on “Testing spike correlation in nonstationary spike data” for the formal use of the joint posterior density for testing the existence of the spike correlation in behaviorally relevant time periods). Second, an EM algorithm developed in the proposed method optimizes the smoothness of the estimated dynamics of the pairwise correlation (i.e., optimization of the hyper-parameters, 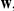 in the state equation, Eq. 8). By the automatic selection of the smoothness parameter, we can avoid the problem of spurious modulation in the estimated dynamic spike correlation caused by local noise, or excessive smoothing of the underlying modulation.

**Figure 2:**
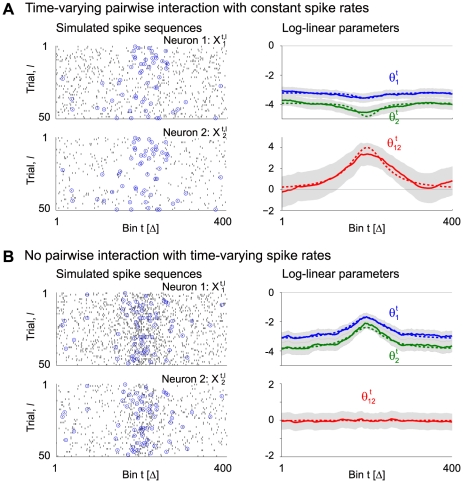
Estimation of pairwise interactions in two simulated parallel spike sequences. (A) Application of the state-space log-linear model to parallel spike sequences with time-varying spike interaction. (Left) Based on a time-dependent formulation of the log-linear model (dashed lines in the right panels represent the model parameters), 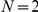 parallel spike sequences, 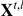, are simulated repeatedly for 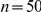 trials (duration: 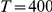 bins). The two panels show dot displays of the spike events of the variables, 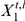 or 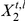 (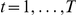 and 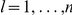). The observed synchronous spike events across the two spike sequences within the same trials are marked by blue circles. (Right) Smoothed estimates of the log-linear parameters, 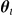 (solid lines, red: pairwise interaction; blue and green: the first order), estimated from the data shown in the left panels. The gray bands indicate the 99% credible interval from the posterior density of the log-linear parameters. The dashed lines are the underlying time-dependent model parameters used for the generation of the spike sequences in the left panels. (B) Application of the state-space log-linear model to independent parallel spike sequences with time-varying spike rates. Each panel retains the same presentation format as in A.

Figure 2A displays an application of our state-space method to 2 parallel spike sequences, 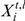 (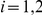, 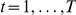, and 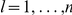), which are correlated in a time-varying fashion. The data are generated as realizations from a time-dependent formulation of a full log-linear model of 2 neurons (Figure 2A left, repeated trials: 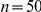; duration: 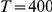 bins of width 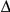). Here, the underlying model parameters, 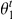, 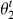, and 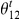 (Figure 2A right, dashed lines), are designed so that the individual spike rates are constant (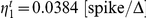 and 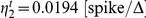), while the spike correlation between the two neurons, 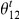, varies in time, i.e., across bins (synchronous spike events caused by the time-dependent correlation are marked as blue circles in Figure 2A left). While the bin-width, 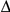, is an arbitrary value in this simulation analysis, the bin-width typically selected in spike correlation analyses is on the milli-second order. If the bin-width is 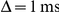, the individual spike rates of simulated neurons 1 and 2 are 38.4 Hz and 19.4 Hz, respectively. The correlation coefficient calculated from these parallel spike sequences is 0.0763. These values are within the realistic range of values obtained from experimentally recorded neuronal spike sequences. By applying the state-space method to the parallel spike sequences, we obtain the smooth posterior density of the log-linear parameters. The right panels in Figure 2A display the MAP estimates of the log-linear parameters (solid lines). The analysis reveals the time-varying pairwise interaction between the two neurons (Figure 2A right, bottom). The gray bands indicate 99% credible intervals from the posterior density, Eq. 9. We used marginal posterior densities, 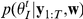 (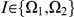), to display the credible intervals for the individual log-linear parameters. The variances of the individual marginal densities were obtained from the diagonal of a covariance matrix of the smooth joint posterior density (Eq. 35).

Figure 2B shows an application of the method to parallel spike sequences of time-varying spike rates. Here, the underlying model parameters are constructed so that the two parallel spike sequences are independent (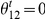 for 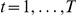), while the individual spike rates vary in time (i.e., across the bins). The observation of synchronous spike events (Figure 2B left, blue circles) confirms that chance spike coincidences frequently and trivially occur at higher spike rates. The analysis based on our state-space method reveals that virtually no spike correlation exists between the two neurons, despite the presence of time-varying rates of synchronous spike events (Figure 2B right, bottom).

#### Simultaneous estimation of time-varying pairwise spike interactions

In this subsection, we extend the pairwise correlation analysis of 2 neurons to the simultaneous analysis of multiple pairwise interactions in the parallel spike sequences obtained from more than 2 neurons.

**Figure 3:**
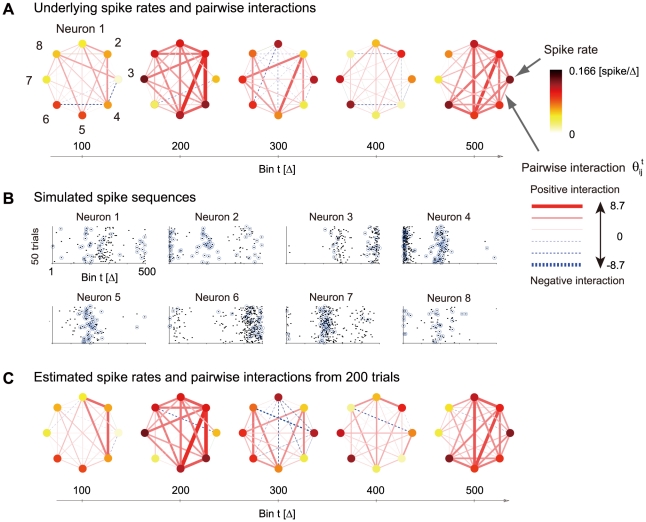
Simultaneous estimation of pairwise interactions of 8 simulated neurons. (A) Snapshots of the underlying model parameters of a time-dependent log-linear model of 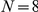 neurons containing up to pairwise interactions (duration: 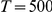 bins) at 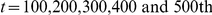 bins. No higher-order interactions are included in the model. Each node represents a single neuron. The strength of a pairwise interaction between the 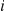
th and 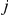
th neurons, 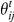 (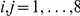), is expressed by the color as well as the thickness of the link between the neurons (see legend at the right of panel B). A red solid line indicates a positive pairwise interaction, whereas a blue dashed line represents a negative pairwise interaction. The underlying spike rates of the individual neurons, 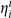 (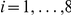), are coded by the color of the nodes (see color bar to the right of panel A). (B) Dot displays of the simulated parallel spike sequences of 8 neurons, 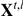, sampled repeatedly for 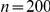 trials from the time-dependent log-linear model shown in A. For better visibility, only the first 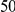 trials are displayed (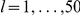). Synchronous spike events between any two neurons (28 pairs in total) are marked by blue circles. (C) Pairwise analysis of the data illustrated in B (using all 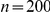 trials) assuming a pairwise model (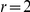) of 8 neurons. The snapshots at the 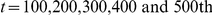 bins show smoothed estimates of the time-varying pairwise interactions, 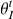 (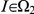), and the spike rates, 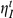 (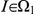). For this estimation, we use 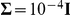 for the prior density of initial parameters. The scales are identical to the one in panel A.

Figure 3 demonstrates an application of our method to simulated spike sequences of 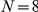 neurons. As an underlying model, we construct a time-dependent log-linear model of 8 neurons with time-varying rates and pairwise interactions (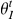, 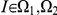, duration: 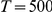 bins). The higher-order log-linear parameters are set to zero, i.e., no higher-order interactions are included in the model. Figure 3A displays snapshots of the dynamics of the parameters of the individual spike rates, 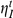 (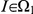), and pairwise interactions, 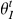 (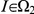), at 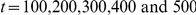. Figure 3B shows the parallel spike sequences (50 out of 200 trials are displayed) simulated on the basis of this model. The spikes involved in the pairwise, synchronous spike events between any two of the neurons (in total: 28 pairs) are superimposed and marked with blue circles. Figure 3C displays snapshots of the simultaneous MAP estimates of the pairwise interactions, 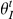 (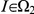), of a log-linear model of 8 neurons applied to the parallel spike train data. In addition, the spike rates were estimated from the dual coordinates, i.e., 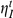 (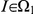). The results demonstrate that the simultaneous estimation of time-varying, multiple pairwise interactions can be carried out by using a state-space log-linear model with up to pairwise interaction terms.

#### Estimation of time-varying triple-wise spike interaction

**Figure 4:**
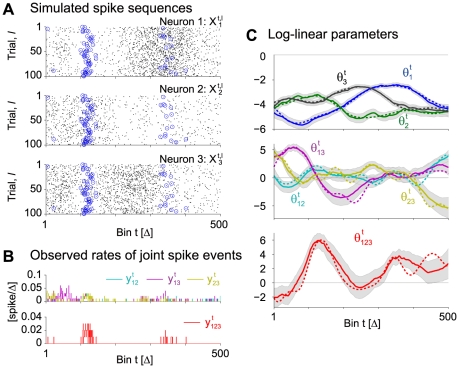
Estimation of triple-wise interaction from simulated parallel spike sequences of 3 neurons. (A) Dot displays of the simulated spike sequences, 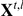, which are sampled repeatedly for 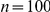 trials from a time-dependent log-linear model containing time varying pairwise and triple-wise interactions (duration: 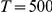 bins; see the dashed lines in C for the model parameters). Each of the 3 panels shows the spike events for each of the 3 variables, 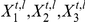 (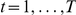 and 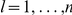), as black dots. Synchronous spike events across the 3 neurons as detected in individual trials are marked by blue circles. (B) Observed rates of joint spike events, 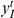 (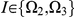). (Top) Observed rates of the synchronous spike events between all possible pair constellations as specified by index 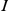 (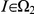). (Bottom) Observed rate of the synchronous spikes across all 3 neurons, 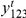. (C) Smoothed estimates of the time-varying log-linear parameters, 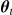. The three panels depict the smoothed estimates (solid lines) of the log-linear parameters, 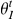, of the different orders (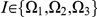), as obtained from the data shown in A and B (top and middle: the first and second order log-linear parameters; bottom: triple-wise spike interaction, 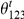). The gray bands indicate the 99% credible interval of the marginal posterior densities of the log-linear parameters. The dashed lines indicate the underlying time-dependent parameters used for the generation of the spike sequences.

Another important aspect of the proposed method is its ability to estimate time-varying higher-order spike interactions that cannot be revealed by a pairwise analysis. To demonstrate this, we apply the state-space log-linear model to 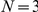 parallel spike sequences by considering up to a triple-wise interaction (i.e., the full log-linear model). Spike data (Figure 4A) are generated by a time-dependent log-linear model (Figure 4C, dashed lines) repeatedly in 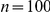 trials. Figure 4C displays the MAP estimates (solid lines) of the log-linear parameters from the data shown in Figure 4A. Here, non-zero parameter 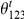 represents a triple-wise spike correlation, i.e., excess synchronous spikes across the three neurons or absence of such synchrony compared to the expectation if assuming pairwise correlations. The gray band is the 99% credible interval from the marginal posterior density, 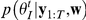, for 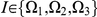.

The credible interval of the higher-order (triple-wise) log-linear parameter in the bottom panel of Figure 4C appears to be larger than those of the lower-order parameters. In general, the observed frequency of simultaneous spike occurrences decreases as the number of neurons that join the synchronous spiking activities increases (note that the marginal joint spike occurrence rate, Eq. 2, is a non-increasing function with respect to the order of interaction, i.e., 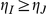 if the elements of 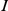 are included in 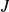). Thus, the estimation variance typically increases for the higher-order parameters, as seen in the bottom panel of Figure 4C. Related to the above, because of the paucity of samples for higher-order joint spike events, the automatic smoothness optimization method selects hyper-parameters that make the trajectories of the higher-order log-linear parameters stiff in order to avoid statistical fluctuation caused by a local noise structure. Given the limited number of trials available for data analyses, these observations show the necessity of a method to validate inclusion of the higher-order interaction terms in the model.

Additionally, we observe that in the later period of spike data (300–500 bins), the dynamics of the estimated triple-wise spike interaction do not follow the underlying trajectory faithfully: The underlying trajectory falls on outside the 99% credible interval. Similar results are sometimes observed when an autoregressive parameter, 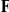, in a state model is optimized (Eq. 8). In contrast, when we replace the autoregressive parameter with an identity matrix (i.e., 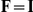, where 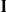 is the identity matrix), the credible intervals become larger. Therefore, such observations do not typically occur. Thus, we also need a method for validating the inclusion of the autoregressive parameter in the state model using an objective criterion. Detailed analyses of these topics will be given in the next section using the example of 3 simulated neurons displayed in Figure 4. In the above example, the state-space log-linear model with an optimized 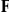 provides a better overall fits to the spike data than the model using 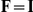, despite an inaccurate representation in part of its estimation. However, for the purpose of testing the spike correlation in a particular period of spike data, we recommend using an identity matrix as an autoregressive parameter, i.e. 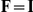, in the state model.

### Selection of state-space log-linear model

For a given number of neurons, 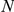, we can construct state-space log-linear models that contain up to the 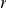
th-order interactions (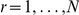). While the inclusion of increasingly higher-order interaction terms in the model improves its accuracy when describing the probabilities of 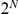 spike patterns, the estimation of the higher-order log-linear parameters of the model may suffer from large statistical fluctuations caused by the paucity of synchronous spikes in the data, leading to an erroneous estimation of such parameters. This problem is known as ‘over-fitting’ the model to the data. An over-fitted model explains the observed data, but loses its predictive ability for unseen data (e.g., spike sequences in a new trial under the same experimental conditions). In this case, the exclusion of higher-order parameters from the model may better explain the unseen data even if an underlying spike generation process contains higher-order interactions. The model that has this predictive ability by optimally resolving the balance between goodness-of-fit to the observed data and the model simplicity is obtained by maximizing the cross-validated likelihood or minimizing the so-called information criterion. In this section, we select a state-space model that minimizes the Akaike information criterion (AIC) [73], which is given as
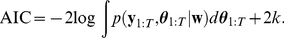
(11)The first term is the log marginal likelihood, as in Eq. 10. The second term that includes 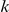 is a penalization term. The AIC uses the number of free parameters in the marginal model (i.e., the number of free parameters in 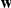) for 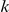. Please see in the Methods subsection ‘Selection of state-space model by information criteria’ for an approximation method to compute the marginal likelihood. Selecting a model that minimizes the AIC is expected to be equivalent to selecting a model that minimizes the expected (or average) distance between the estimated model and unknown underlying distribution that generated the data, where the ‘distance’ measure used is the Kullback-Leibler (KL) divergence. The expectation of the KL divergence is called the KL risk function.

#### Selection from hierarchical models

Here, we examine the validity of including higher-order interaction terms in the model by using the AIC. We apply the model selection method to the spike train data of 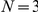 simulated neurons. The data are generated by the time-varying, full log-linear model that contains a non-zero triple-wise interaction terms shown in Figure 4C (dashed lines). The AICs are computed for hierarchical state-space log-linear models, i.e., for models of interaction orders up to 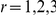. To test the influence of the data sample size on the model selection, we vary the number of trials, 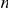, used to fit the hierarchical log-linear models. The results are shown in Table 2 for 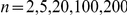. For a small number of trials (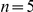), a model without any interaction structure (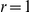) is selected. For larger numbers of trials, models with larger interaction orders are selected. For 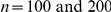, the full log-linear model (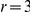) is selected.

**Table 2:** AICs for different numbers of trials.

The number of trials	Model order		
	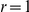	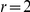	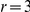
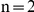	775.15*	807.146	841.96
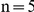	1957.4	1890.6*	1922
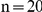	7857.2	7565.1*	7585.8
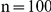	37791	36264	36231*
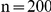	75443	72366	72283*

This table displays the AICs of a state-space log-linear model with increasing interaction orders: an independent model (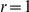), pairwise model (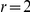), and full model (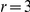), applied to simulated 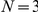 spike sequences with an increasing number of trials, 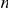, considered in the analysis. The spike data are identical to that shown in Figure 4A. The asterisk indicates the model that minimizes the AIC.

Below, we examine whether the AIC selected a model that minimizes the KL risk function by directly computing its approximation using the known underlying model parameters. First, Table 3 shows how often a specific order is selected by the AIC by repeatedly applying the method to different samples generated from the same underlying log-linear parameters (Figure 4C, dashed lines). We examine two examples: One in which a sample is composed of 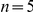 trials (left) and the other of 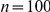 trials (right). We repeatedly compute the AICs of state-space models of different orders (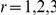) applied to 100 data realizations (of the respective number of trials). We then count how often a model of order 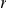 is selected by minimizing the AIC. For comparison, the table includes the outcomes from other criteria such as the Bayesian information criterion (BIC) [79], [80] and the predictive divergence for indirect observation models (PDIO) [81], which are suggested for models containing latent variables. Please see the Methods section for the details of these criteria. Next, Table 4 displays the most frequently selected model (from 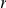 = 1,2,3) by the various information criteria when they are applied to 100 data realizations as a function of the number of trials in each data set, 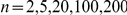 (see Table 3 for the outcomes of 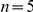 and 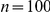).

**Table 3 pcbi-1002385-t003:** Models selected using different information criteria.

	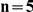 trials			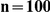 trials		
	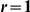	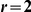	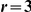	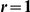	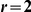	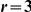
AIC	29	71*	0	0	3	97*
BIC	15	85*	0	0	99*	1
PDIO	46*	25	29	3	60*	37

The state-space log-linear models containing interactions up to the 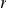
th-order (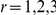) are applied to the data from 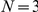 simultaneous spike sequences. The spike data is generated from a time-dependent full log-linear model (see dashed lines in Figure 4C) repeatedly for 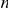 trials; either 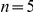 (left) or 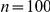 (right) trials. For this data set, we compute three information criteria (AIC, BIC, and PDIO) and find the order of the model that minimizes these information criteria. We repeated the selection of the model order 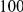 times, using each of the criteria and using the spike data that contains respective number of trials (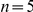 or 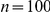). The count of the order of spike interactions that minimizes the applied information criteria is increased by 1, and finally expressed as a frequency. The asterisk marks the most frequently selected model.

**Table 4:** Model orders selected by different information criteria for different numbers of trials.

	The number of trials				
	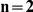	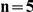	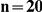	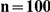	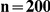
AIC	1	2	2	3	3
BIC	2	2	2	2	3
PDIO	1	1	1	2	2
KL-risk	1	2	3	3	3
MSE	1	1	3	3	3

The state-space log-linear models of different orders (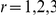) are applied to samples of the 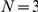 spike sequences of 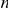 repeated trials generated from a time-dependent full log-linear model (indicated by the dashed lines in Figure 4C). Three data-driven information criteria, AIC, BIC, and PDIO, are computed for the fitted state-space models of the different orders, 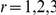. The count for the model order 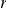 that minimizes the respective criteria is determined by repeating the process for 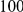 repetitions as in Table 3. The most frequently selected model order, 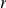, is displayed for each of the information criteria and for the different numbers of trials, 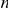. For comparison, we also show the order of interactions that minimizes the KL risk function (KL-risk) and mean squared error (MSE). We approximated the KL-risk and MSE as follows. At each bin, we compute the KL-divergence (Eq. 21), between a full underlying log-linear model of 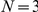 neurons and the estimated log-linear model whose parameters are given by the MAP estimates of the 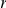 th-order model. The total sum of the all KL-divergences from 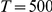 bins is used as the distance between the two (time-dependent) models: i.e., 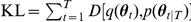, where the function 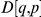 is given in Eq. 21. 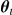 represents the underlying log-linear parameters used to generate the data. 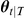 is its estimate from one sample composed of 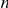 trials. The parameters higher than the 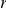 th-order that are not included in the model are set to zero. The KL-risk function is estimated as the average of the KL-divergences of 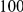 realizations of the spike data, each composed of 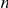 trials. To obtain the MSE, we first computed the sum of the squared errors (SE) : 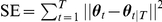, using one sample composed of 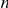 trials. The MSE is then estimated as the average of the SEs over 100 samples.

We compare the outcomes of the AIC and two other information criteria with the model order that minimizes the KL risk function (KL-risk). For this goal, we include in Table 4 the model order that minimizes the KL-risk between the underlying log-linear model (Figure 4C, dashed lines) and estimates of the 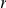 th-order model. In addition to the KL-risk, we calculate the mean squared error (MSE) between the underlying model parameters and corresponding estimates. Please see in the caption of Table 4 how we compute the KL-risk and MSE for this analysis. We find that the KL-risk and MSE select the same model, except for the case of 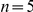 trials. In comparison to the KL-risk, the BIC tends to select models with an excessively higher-order of interaction (over-fitting) for a small number of trials (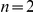) and tends to choose lower-order models for 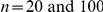. The PDIO mostly selects lower-order models. In contrast, the AIC follows the selection of the KL-risk minimization principle, except for 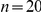, where it shows a conservative choice compared to the KL-risk.

We repeat the same analysis for spike data generated from an underlying model that contains up to pairwise interactions, but does not contain the triple-wise interaction term. The purpose of this analysis is to show that the methods do not select models with excessively higher orders of interaction than those actually contained in the data. To construct such an underlying model, we project the full model (shown in Figure 4C) onto the subspace of a pairwise log-linear model, 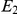. The projection model does not contain any triple-wise correlation, while the 1st and 2nd order expectation parameters (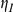 for 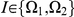) are the same as those of the full model that was used to generate the data in the analysis of Tables 2–4. Table 5 displays the most frequently selected model orders by the AIC, BIC, PDIO, along with the selections by the KL-risk and MSE. We find that the pairwise model is the most frequently selected model under all of the criteria for the samples with a large number of trials (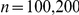). Under this condition, only the AIC among the other data-driven methods follows the KL-risk selection. These results lead us to the conclusion that the AIC is a reliable measure to assess the goodness of fit of the state-space log-linear model.

**Table 5:** Model orders selected using different information criteria for model without triple-wise spike interaction.

	The number of trials				
	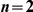	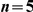	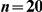	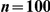	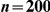
AIC	1	2	2	2	2
BIC	2	2	2	2	2
PDIO	1	1	1	2	2
KL-risk	1	2	2	2	2
MSE	1	2	2	2	2

Similar to Table 4, the state-space log-linear models (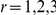) are repeatedly applied to 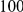 samples of 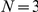 spike sequences with 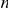 trials. In contrast to Table 4, here a sample of spike sequences is generated from a pairwise (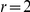) time-dependent log-liner model of 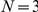 neurons. This underlying model is a projection of the full log-liner model examined in Table 4 (dashed lines in Figure 4C) to the pairwise model space. The frequencies of the 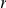 th order model that minimizes AIC, BIC, and PDIO are counted by repeatedly applying models with different orders (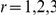) to 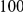 samples (each with 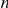 trials). The most frequently selected models are displayed for the different criteria and for different numbers of trials, 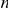, as in Table 4. The rows for the KL-risk and MSE display the models that minimize the estimates of the KL-risk and MSE.

#### Selection of state transition model

In addition to validating the inclusion of the order of spike interactions, we examine state models (Eq. 8) with different conditions for the hyper-parameters by the AIC, using as an example the same spike train data of 3 neurons with 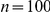 trials (displayed in Figure 4A). The tested state models are (i) a time-independent model in which the hyper-parameters are fixed as 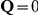 and 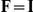, where 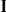 is an identity matrix; (ii) a random walk model (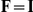), in which only the covariance matrix, 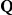, is optimized via the EM algorithm; and (iii) a 1st-order autoregressive model, in which 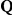 and 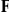 are both optimized. With these settings, the state-space log-linear model of case (i) becomes a stationary log-linear model, which has been frequently employed in spike data analyses, often in the form of a maximum entropy model [55]–[57], [82]. In contrast, the state-space models of cases (ii) and (iii) are nonstationary because the joint distribution of the spike pattern changes in time as a result of the time-varying log-linear parameters. The interaction parameters themselves are assumed to be nonstationary in the state model in case (ii), and can be either stationary or nonstationary in the state model in case (iii). To see this, let 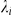 be the eigenvalues of 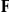, i.e., the solutions for 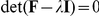. For a stationary process, the eigenvalues have to satisfy 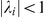, otherwise the process is nonstationary. This excludes the case 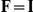.

The AICs of the full model with the various state-equations become progressively smaller for increasingly complex state-space models (38525 for case (i), 36263 for case (ii), and 36231 for case (iii)). The fact that conditions (ii) and (iii) are better fitted to the data than condition (i) confirms that the proposed method of time-varying spike correlation analysis performs better than an analysis based on a stationary log-linear model for this data. In addition, the fact that condition (iii) better fits the data than condition (ii) supports the use of autoregressive models (note: The estimation in Figure 4B is done with state model (iii)). We find that the fitted 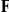 (to the data in Figure 4A) yields an eigenvalue larger than 1, indicating that the underlying state process (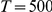 bins) is modeled as a nonstationary process. We consider that the nonstationary state model is selected because of the relatively short observation period, during which a nonstationary trend for the parameters can appear even if the state process is stationary in the long run.

### Test for presence of spike correlation in nonstationary spike sequences

One of the goals of a time resolved analysis of spike correlation is to discover dynamical changes in the correlated activities of neurons that reflect the behavior of an animal. This implies the necessity of dealing with the within-trial nonstationarity that is typically present in the data from awake behaving animals. However, we know from other correlation analysis approaches that, if not well corrected for, nonstationary spike data bear the potential danger of generating false outcomes [25], [83]. Here we deal with the within-trial nonstationarity of the data by using the state-space log-linear model while assuming identical dynamic spiking statistics across trials (across-trial stationarity). In order to correctly detect the time-varying correlation structure within trials, we apply to the state-space log-linear model a Bayesian model comparison method based on the Bayes factor (BF) [74]–[76], and combine it with a surrogate approach. The BF is a likelihood ratio for two different hypothetical models of latent signals, e.g., in our application, different underlying spike correlation structures. Using the BF, we determine which of the two spike correlation models the spike data supports. By computing the BF for a particular task period in a behavioral experiment, we can test whether the assumed correlation structure appears in association with the timing of the animal's behavior. In the following, we denote a specific task period of interest by the time period 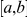.

In this study, the BF, 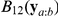, is defined as the ratio of the marginal likelihoods of the observed spike patterns, 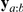, in the time period 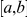 under different models, 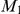 or 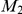, assumed for the hidden state parameters,
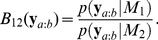
(12)By successively conditioning the past, the BF is computed by the multiplication of the bin-by-bin one-step BF given at time 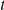 as 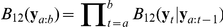. Here, the bin-by-bin BF at time 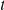, 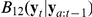, can be calculated as (see the Methods subsection, ‘Bayesian model comparison method for detecting spike correlation’),
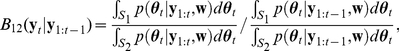
(13)where 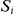 is the space of the interaction parameters, 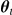, for the model, 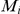 (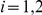). In Eq. 13, 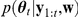 is the filter density and 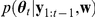 is called the one-step prediction density, both of which are obtained in the Bayesian recursive filtering algorithm developed in the Methods section (cf. Eqs. 25, 26 and Eqs. 31, 32). Therefore, the bin-by-bin BF at time 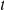, 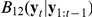, is the ratio of the odds (of opposing models) found by observing the spike train data up to time 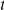 (filter odds, the numerator in Eq. 13) to the odds predicted from 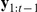 without observing the data at time 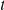 (prediction odds, the denominator in Eq. 13). Thus, an unexpected synchronous spike pattern that significantly updates the filter odds for the interaction parameters from their predicted odds gives rise to a large absolute value for the BF. Because the posterior densities are approximated as a multivariate normal distribution in our filtering algorithm, the BF at time 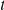 can be easily computed by using normal distribution functions. Please see the subsection, ‘Bayesian model comparison method for detecting spike correlation’, in the Methods section for the derivation of Eq. 13 and detailed analysis of the BF.

The BF becomes larger than 1 if the data, 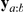, support model 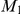 as opposed to 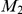 as an underlying spike correlation structure and becomes smaller than 1 if the data support model 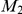 as opposed to 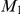. Alternatively, it is possible to use the logarithm of the BF, known as the ‘weight of evidence’ [75] which becomes positive if the data support model 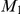 as opposed to model 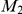 and negative in the opposite situation. Below, we display the results for the BF in bit units (logarithm of the BF to base 2), i.e., the weight of evidence. By sequentially computing the bin-by-bin BF, we can obtain the weight of evidence in a period 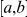 as the summation of the local weight of evidence: 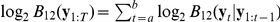.

An intuitive interpretation of the BF values is provided in the literature [74], [76]. For example, in [76], a BF (weight of evidence) from 1.6 to 4.3 bit was interpreted as ‘positive’ evidence in favor of 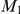 against 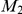. Similarly, a BF from 4.3 to 7.2 bit was interpreted as ‘strong’ evidence, and a BF larger than 7.2 bit was found to be ‘very strong’ evidence in favor of 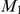 against 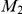. While the classical guidelines are useful in practical situations, they are defined subjectively. Thus, in this study, in order to objectively analyze the observed value of the BF, we combine the Bayesian model comparison method with a surrogate approach. In this surrogate method, we test the significance of the observed BF for the tested spike interactions by comparing it with the surrogate BFs computed from the null-data generated by destroying only the target spike interactions while the other structures such as the time-varying spike-rates and lower-order spike interactions are kept intact.

The BF in a behaviorally relevant sub-interval 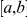 can be computed from the optimized state-space log-linear model fitted to the entire spike train data in 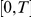. Here, for the purpose of testing spike correlation in the sub-interval, we recommend to use 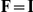 in the state model because the autoregressive parameters are optimized for entire spike train data, which are not necessarily optimal for the sub-interval. Similarly, a typical trial-based experiment is characterized by discrete behavioral or behaviorally relevant events, e.g. movement onset after a go signal or a cue signal for trial start, etc. Thus, on top of the expected smooth time-varying change in the spike-rate and spike-correlation, sudden transitions may be expected in their temporal trajectories. Because we use time-independent smoothing parameters (i.e., hyper parameter 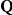 in Eq. 8) that were optimized to entire data (see the EM algorithm in the Methods section), such abrupt changes may not be captured very well. This may cause a false detection or failure in the detection of the spike correlation at the edge of a task period. For such data, we suggest applying the Bayesian model comparison method to state-space models which are independently fitted to each of the task periods (or relatively smooth sub-intervals within each task period).

#### Detecting triple-wise spike correlation in simulated nonstationary spike sequences

**Figure 5:**
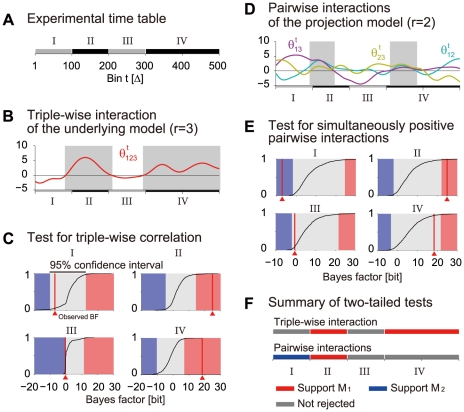
Detection of spike correlations and their relation to pseudo experimental protocol (simulation study). (A) Sketch of an assumed experimental time course composed of four epochs (I–IV), e.g., of different behavioral task conditions. Epochs I–III have a duration of 100 bins, period IV has a duration of 200 bins. (B) Time-varying triple-wise spike interaction parameter of the underlying model (cf. Figure 4C, 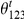) used for the simulation of spike data (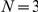 neurons, 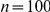 trials) during the time course outlined in A. The gray areas indicate the time intervals in which the triple-wise interaction is positive: 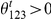. (C) Hypothesis testing for a triple-wise spike correlation based on surrogates. In each time period (I–IV), we perform a test on the Bayes factor (BF) resulting from the original data. The observed BF (Eq. 12), marked by a red line and triangle, is computed as evidence of a positive triple-wise interaction, 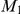: 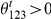, as opposed to a zero or negative triple-wise interaction, 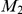: 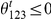. We then compare the ‘observed BF’ with cumulative distribution functions (CDFs, solid lines) for the BFs derived from surrogate data sets generated from a model containing only up to pairwise interactions (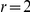). In all of the CDFs, the gray area indicates the 95% confidence interval of the distribution. If the observed BF falls into the lower tail of the distribution (blue area), 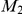 is supported; if it falls into the upper tail (red marked area), 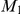 is supported. (D) Time courses of the underlying pair-interaction parameters of a pairwise log-linear model (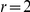), 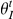 (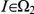). These underlying parameters are obtained by projecting the full underlying model (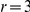) in Figure 4 to the pairwise model space, 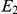. The gray areas indicate the time periods in which all of the pairwise interactions are positive. (E) Similar tests as in C, but for the BF computed as evidence for the presence of simultaneous positive pairwise interactions, 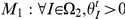, as opposed to the absence of such an assembly, 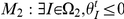. (F) Compact visualization of the test results from C and E. The colored bars show which hypotheses are supported in the different time segments (red and blue) and where the null hypothesis cannot be rejected.

In this subsection, we examine a method to test for the presence of a higher-order (triple-wise) spike correlation using simulated spike train data with known spike interaction dynamics. In this context, we again use the simulated spike data of 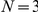 neurons (of length 500 bins with 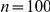 trials) shown in Figure 4A, which was generated by the time-varying model, as shown in Figure 4C (dashed lines). In the following, we assume for this data an underlying experimental protocol that can be segmented into behaviorally-relevant time periods, I–IV, as indicated in Figure 5A, as for example a period before the trial starts, a preparatory period, a movement period, etc. (e.g., see [8], [19] for a typical experimental protocol).

In each of the time periods, we compare opposing models for the hidden log-linear parameters of 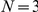 neurons, using a full model (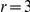). In one hypothetical model, 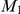, we assume that the triple-wise interaction term is positive, 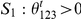. The time (bin) index 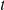 expresses that the BF is computed under the same models for all of the time steps, 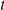, in the respective time period. In the other model 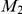, we assume that the triple-wise interaction term is smaller than or equal to zero, 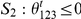. Neither model makes specific assumptions about the first and second order log-linear parameters; thus, they are allowed to be real numbers. These parameters are integrated out in Eq. 13. In this simulation study, we independently applied a state-space log-linear model to each of the four periods and computed the respective BFs. Figure 5B displays the ground truth of the time-dependent triple-wise interaction parameter, 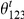, of the full log-linear model. The gray areas indicate periods where the triple-wise spike interaction, 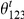, is positive: Model 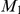 is true in this period. In the remaining periods (white), the model of a negative triple-wise spike interaction, 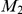, is true.

We compare the observed BF for the tested correlation models to the BFs computed from surrogate data sets resampled under a null hypothesis of no tested order of spike interaction. Specifically, to test the existence of a triple-wise spike correlation, the surrogate BFs are computed from resampled spikes generated from a model containing no triple-wise spike interaction. For this, we first apply a pairwise state-space log-linear model (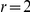) to the observed spike data to derive estimates of the time-varying spike rates and dynamic pair-interactions. Then we generate 1000 surrogate samples of 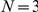 parallel spike sequences, where each sample is composed of 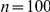 trials, using the fitted pairwise state-space log-linear model. Thus, the resampled spike sequences exhibit the same time-varying spike rates and pairwise correlations as the original data, but the triplet coincidences occur on a chance level, as expected by the individual spike rates and pairwise correlations among the neurons. The surrogate BFs of a triple-wise spike correlation (using 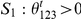, 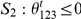) are computed for each surrogate data set by applying the full model (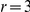).

Figure 5C shows the observed BF (red vertical line and red triangle) and the cumulative distribution function (CDF) of the surrogate BFs (solid black lines) for each of the four time periods, I–IV. In each of the graphs, the gray area indicates the 95% confidence interval derived from the distribution of 1000 respective surrogate BFs. If the observed BF falls outside the confidence interval, the null hypothesis that no tested order of spike interaction exists is rejected. Further, by the two-tailed test, if the observed BF falls outside the confidence interval into the red area of the distribution on the right (i.e., significantly positive BF values), the data supports model 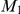 as an underlying correlation structure, whereas if it falls into the blue area on the left, we conclude that it is a significantly negative BF, i.e., 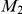 is supported. If the observed BF falls into the 95% confidence interval, the null hypothesis that no tested order of spike correlation exists is not rejected. We first look at the results for periods II and IV in Figure 5C. In these periods, the null hypothesis of no triple-wise spike correlation is rejected, and the observed BF correctly suggests a positive triple-wise correlation model (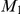), reflecting the fact that the underlying triple-wise interaction term is positive throughout the two periods (see periods II and IV in Figure 5B). In contrast, in periods I and III, where a negative triple-wise correlation model (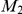) is true, the null hypothesis of no triple-wise correlation cannot be rejected.

It is possible that the synchronous spike events observed among multiple neurons are detected as simultaneous increases in pair interaction terms using the log-linear model of the second order (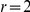), without having to take higher-order spike interactions into consideration. Paradoxically, this issue becomes relevant when a triple-wise spike correlation is a dominant factor in the generation of synchronous spike events because the projection of a full model (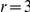) that contains a positive triple-wise interaction term to a pairwise model induces simultaneous increases in the pair interaction terms in that projected model (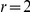). In such a case, an analysis of the higher-order spike correlations might just add redundant information about an animal's behavior to lower-order analyses. Thus, we repeat an analysis similar to the one above under this alternative hypothesis, using a model that contains up to pairwise interactions (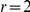). In this test, the BF (Eq. 12) is computed using the following assumed models on the hidden log-linear parameters: 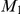, all three neurons simultaneously exhibit positive pairwise interactions (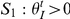 for all 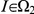); and 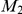, at least one of the pairwise interactions is not positive (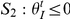 for at least one 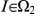). The first order log-linear parameters are again assumed to be real values. Surrogate data sets are obtained by resampling spike sequences from the first order state-space log-linear model (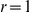) fitted to the data. The surrogate BFs for the model of simultaneously positive pairwise interactions, as opposed to its complementary model, are computed for each of the surrogate data sets by applying a pairwise model (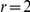). Figure 5D displays the ground truth of the dynamics of the pairwise interaction terms, 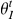 (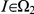), of the pairwise log-linear model. These are obtained by projecting the full underlying model (Figure 4C dashed lines) to the probability space of the second-order model (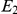). Note that in period II, the pair-interaction terms simultaneously increase because the effect of the triple-wise interaction in 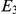 is projected to this sub-space model (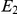) (see Figure 4C, middle panel, for comparison, and observe that there is no such increase in the pair-interaction terms of the full log-linear model). The gray areas indicate the period where model 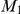 (simultaneously positive pairwise interactions) is true. Figure 5E displays the results for testing the simultaneous increases in the pair interactions. We again look at periods II and IV. In period II, the null hypothesis of independent spike sequences is rejected, and model 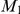 is supported, reflecting the fact that the projected pairwise interactions are positive for most of this time period. In contrast, the same null hypothesis cannot be rejected in period IV because neither model 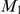 nor 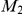 alone can support the data in this period: Models 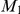 and 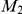 are true in the first and second halves of period IV, respectively (see Figure 5D).

Figure 5F summarizes the results for periods I–IV using bars in corresponding colors. The results of our tests reflect the dynamics of the underlying parameters in Figures 5B and D, and we find that in period IV, only the test for a triple-wise spike correlation detected the presence of interactions among all three neurons in the data. This is because, in this time period, the dynamics of the underlying triple-wise interaction correlate well with the assumed behavioral time table, whereas the dynamics of the simultaneous pairwise interactions do not. In summary, it is possible that the higher-order analysis allows us to discover the correlated activities of multiple neurons associated with behavioral events, which may not be revealed by pairwise analyses.

#### Detecting triple-wise spike correlation in neural spike data from behaving monkey

**Figure 6:**
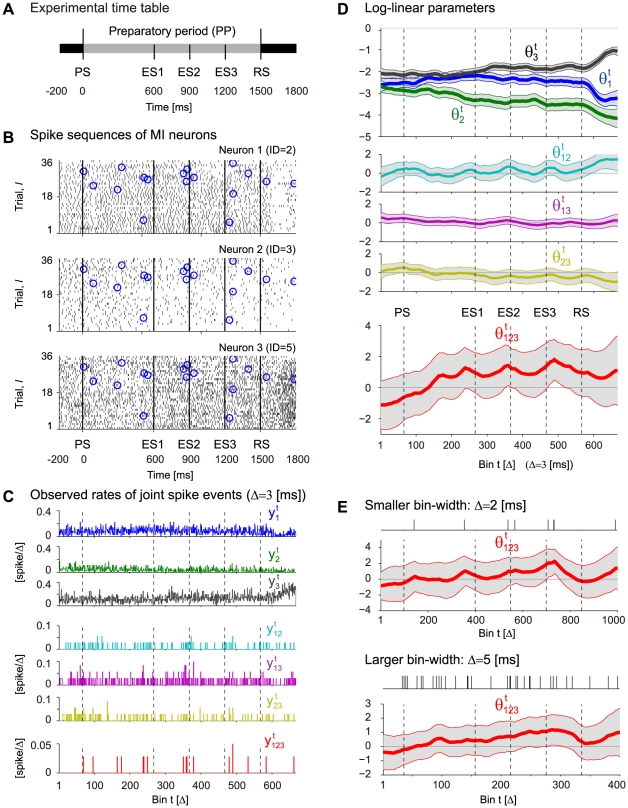
Analysis of experimental spike data using the state-space log-linear model. (A) Experimental time table of delayed-response hand movement task. The experiments were designed and conducted by Riehle and her colleagues [8], [84]. During the preparatory period (PP, 1500 ms) that starts with the preparatory signal (PS), the presentation of the response signal (RS) was expected at three distinct moments at 600, 900, and 1200 ms (expected signals, ESs). Here the RS occurs finally after the longest possible delay of 1500 ms. After the RS, the requested movement was executed (reaction and movement time, RT-MT). See [8], [84] and the text for the detailed experimental protocol. (B) Dot displays of the spike sequences (duration: 2 s, sampling resolution 1 ms) of three neurons simultaneously recorded from the primary motor cortex (MI). The spike sequences are aligned at the onset of the PS (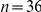 trials). The synchronous spike events across the 3 neurons detected in individual trials (detection in bins of 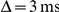 width) are marked by blue circles. (C) Observed rates of joint spike events, 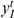 (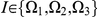). All of the events are detected in bins with a width of 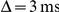. (Top) Observed rates of the spike occurrence of the individual neurons (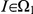). (Middle) Observed rates of the synchronous spike events between two neurons specified by index 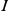 (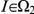). (Bottom) Observed rate of the synchronous spike events of all 3 neurons, 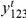. (D) Estimation of the time-varying log-linear parameters of 3 neurons, 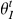 (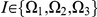), from the binary data shown in C, according to the method summarized in Table 1. The panels depict the MAP estimates of the log-linear interaction parameters (solid lines; from top to bottom, the first and second order log-linear parameters and a triple-wise spike interaction, 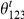). The gray bands indicate the 95% credible interval. In this analysis, we used an identity matrix as an autoregressive parameter, i.e. 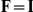, in the state model. The covariance matrix of the initial parameter was fixed to 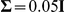. (E) The smoothed estimate of a triple-wise spike interaction, 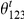, is computed from binary data constructed using a bin-width of 2 ms (Top) and a bin-width of 5 ms (Bottom). The top of each panel shows the timing of the synchronous spike events of all three neurons.

Finally, we demonstrate the application of our method on simultaneous spike recordings from the primary motor cortex (MI) of an awake, behaving monkey. The data were recorded by Alexa Riehle and her colleagues [8] to test the hypothesis that neuronal cooperativity is involved in the planning of motor actions. Therefore, Riehle et al. designed a behavioral task in which different durations of preparation intervals were provided to the monkeys before they had to perform an arm movement and touch a target on a screen. The detailed time table of the task is as follows (cf. Figure 6A). A trial started by a signal indicating to the monkey that he may initiate the task by pressing a button. After initiating the trial, the monkey had to wait for 1000 ms until a preparatory signal (PS) was delivered, indicating to the monkey that now the preparation interval started. After appearance of a response signal (RS) at 600, 900, 1200, or 1500 ms (randomly selected with equal probability), the monkey had to move his arm and touch the target position. The reaction times of the monkey showed a dependence on the duration of the preparatory period (PP): The longer the PPs the shorter the reaction times [8], [19], [84]. The conditional probability for the occurrence of the RS increases during the longer PP, in particular at the times when the RS may occur. The hypothesis for the underlying network activity realizing reduced reaction times with longer PPs was that the system is better prepared for the requested movement by increasingly enhancing the synchrony in the relevant network to facilitate the response. Indeed, Riehle et al. showed that cortical neurons modulate the degree of excess synchronous spiking activities independently from their individual firing rates in conjunction with the occurrence of the expected RSs [8], [19], [84]. An open question that could not be conclusively answered in that study was if larger groups of neurons than pairs coordinate their activity in relation to the expected events. Here, we approach this question by applying our newly developed method and will indeed demonstrate the existence of higher-order (triple-wise) spike interaction in relation to the behavioral task.

To that end, we analyze data which were in part analyzed by the Unitary Events analysis method (neuron id 2 and 3; shown in Figure 2 in [8]). Figure 6B shows this particular data set that consists of three simultaneously recorded neurons (neuron id 2, 3, and 5; Here we denote them as neuron 1, 2, and 3) observed during trials (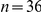) of the longest preparatory period of 1500 ms. As in Riehle et al. (Figure 2 in [8]) we align the trials at the PS, and analyze the data for the interaction parameters as a function of time from 200 ms before the PS and 1800 ms after the PS. This time segment is composed of three behavioral epochs: an interval before the PS (200 ms), the PP with three expected signals (ESs, at 600, 900 and 1200 ms), and a period after the RS at 1500 ms including the reaction time and partly the movement (reaction and movement time, RT-MT) (see Figure 6A). The average firing rates of the neurons during the PP are 29.4, 12.9, and 41.9 Hz for neurons 1, 2, and 3 (neuron id 2, 3, and 5) respectively. We constructed binary sequences, 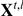, from the spike times of the neurons by binning the data using a bin-width of 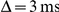. Figure 6C displays the occurrence rates for the individual spiking activities (upper panel) and the pair and triple synchronous spike events (middle and bottom panels) detected in the bins with a width of 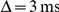.

We apply a full log-linear model of 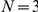 neurons with up to a triple-wise interaction term to the binary spike data shown in Figure 6C. Figure 6D displays the estimated dynamics of the interaction parameters in the log-linear model by using the method summarized in Table 1. The thick bold lines indicate the MAP estimates, i.e., the most probable paths, of the interaction parameters. The gray bands and thin solid lines mark the 95% credible interval (the Bayesian analogue of the confidence interval) computed from a marginal posterior density. The last parameter of the log-linear model, 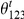, indicates the triple-wise interaction among the three neurons in the MI. Strong positive (or negative) value of this term indicates that the three neurons are triple-wise correlated, i.e. dependent in a manner that cannot be explained by pair correlations of the neurons. Specifically, a positive triple-wise interaction value means that the occurrence of the synchronous events among the three neurons is more frequent than the chance level expected from the observed individual rates and pairwise spike correlations among them.

The analysis of the MI neurons using the state-space log-linear model reveals that the triple-wise interaction, 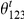, during the PP gradually increases, with additional local peaks at the ESs that also increase in heights towards the end of the PP (Figure 6D, bottom panel). This result is consistent with the results found by Riehle and collaborators for a subset of the neurons of the same data set (Figure 2 in [8]). In this previous study neuron 1 and 2 (neuron id 2, 3) were analyzed for excess spike synchrony using the Unitary Events analysis [32], [33]. The analysis revealed that the two neurons exhibit a modulation of significant excess spike synchrony, with peaks at the ESs (despite the first) and at the RS.[8], [32], [84]. That observation was interpreted as evidence that the neurons cooperate to prepare for motor action and facilitate the efficiency for movement execution [8], [84]. Similarly, occurrences of synchronous spike events of the three neurons roughly coincide with the expected events (ESs) (Figure 6C, bottom panel); accordingly, our result shows that the triple-wise interaction, 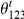, is also locked to the ESs, however decreases at RS. Note that the triple-wise interaction is not only determined by the frequency of synchronous spike events of all three neurons but is also determined by frequencies of other observed spike patterns across trials. However, for typical neural spike train data with low spike rates and low rates of synchronous spiking of pairs of neurons, the occurrence of synchronous spike events of three neurons can significantly increase the triple-wise interaction. In the subsection ‘Bayesian model comparison method for detecting spike correlation’ in the Methods section we explore the contribution of different spike patterns in different spiking scenarios to the evidence of a triple-wise spike correlation in simulated data. Figure 6E displays modulation of the interaction term, 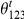, using the binary data constructed with bins of smaller (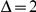 ms, upper panel) and larger (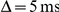, lower panel) widths. The top of each panel shows the timing of the synchronous spike events of all three neurons. The sample sizes for synchronous spike events across all three neurons for the 2 ms bin-width are greatly reduced from those observed for a bin-width of 3 ms. Thus estimated dynamics is much less structured: The data prevents us from detecting existence of a triple-wise spike correlation using the proposed statistical test (see below for the application of the test method). With larger bins of a width of 5 ms, the precise locking of the synchronous spike events across the three neurons to ESs is no longer apparent. However, the state-space log-linear model reveals a gradual increase in the strength of the triple-wise interaction until the end of the PP.

**Figure 7:**
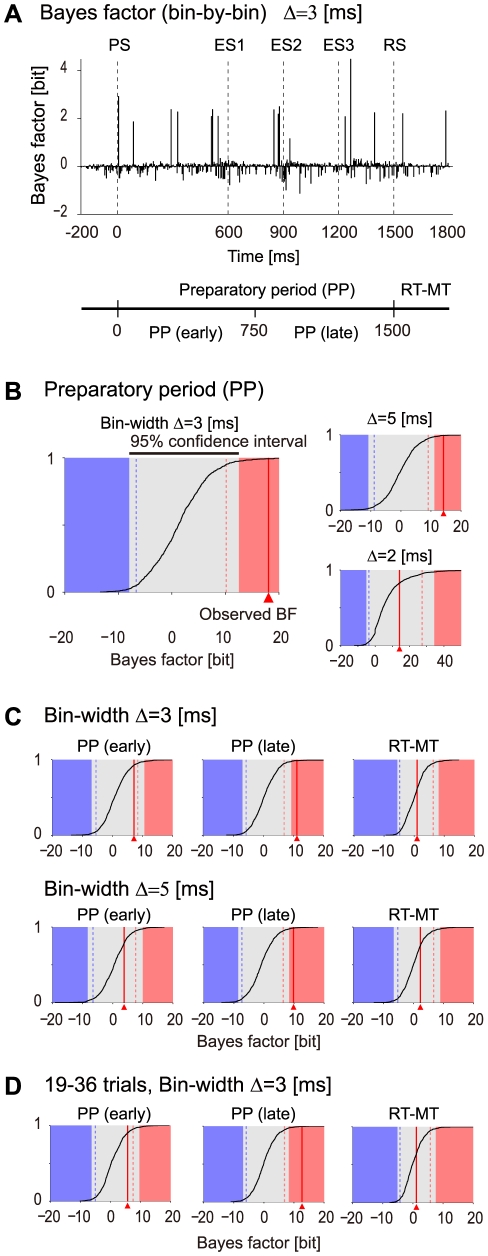
Detection of triple-wise spike correlation of MI neurons. (A) (Top) The bin-by-bin Bayes factor (BF) for a model of triple-wise spike interaction computed locally in time in bit units, Eq. 13. The bin-by-bin BF is computed from Eq. 13, using the state-space log-linear model fitted to the spike data (a total of 36 trials, 2 s binned using 3 ms bin-width; cf. Figure 6C). The BF computes evidence of a positive triple-wise spike interaction, 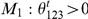, as opposed to a zero or negative triple-wise interaction, 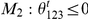. The evidence for model 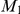 as opposed to 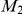 in a behaviorally relevant time period is obtained by summing the log of the bin-by-bin BF in that period (cf. Eq. 12). (Bottom) Two behavioral periods (preparatory period, PP, and reaction and movement time, RT-MT) tested for presence of a triple-wise spike correlation. In addition, to examine the evolution of the triple-wise spike correlation in the PP, the PP is divided into early and late stages at the middle of the PP. (B) (Left) The observed BF for an entire PP (marked by a red line and triangle) computed using Eq. 12 (bin-width: 3 ms). We then test the ‘observed BF’ using a distribution function (solid line) of null BFs derived from surrogate data sets generated from a fitted model containing only up to pairwise interaction terms (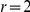). The gray area indicates the 95% confidence interval of the distribution. Vertical dashed lines indicate the 90% confidence interval. If the observed BF falls into the lower tail of the distribution (blue area), 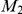 is supported, if it falls into the upper tail (red marked area), 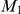 is supported. (Right) The same analysis in the left panel, but with binary data constructed using larger (5 ms, upper panel) and smaller (2 ms, lower panel) bin-widths. (C) (Top) Test of observed BFs (bin-width 3 ms) computed in distinct periods: early PP (0–750 ms), late PP (750–1500 ms), and RT-MT (1500–1800 ms). (Bottom) The same test as in the top panel, except that binary data using a bin-width of 5 ms were used. (D) Test of observed BFs (bin-width: 3 ms) in each period using spike data from only the last half of the 36 trials (trials 19–36).

To strengthen the findings that a triple-wise interaction term increases during the PP, we test for the presence of a triple-wise spike correlation in the PP using the Bayes factor (marginal likelihood ratio, Eq. 12). We compute the BF for the opposing models using a full model (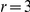) of 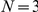 neurons for the hidden log-linear parameters. In one hypothetical model, 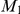, we assume that the triple-wise interaction term is positive, 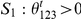, in the other model, 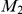 we assume that the triple-wise interaction term is smaller than or equal to zero, 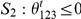. A large positive value of the BF indicates that the spike data in that period supports model 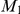 as opposed to model 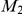, i.e., the existence of excess synchronous spike events among the three neurons in comparison with the chance rate given by spike rates and pairwise correlations. A large negative value of the BF shows support of model 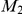 as opposed to model 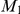, i.e., a paucity of synchronous spikes for the three neurons. Figure 7A shows the BF computed bin-by-bin (Eq. 13) in bit units. In the bottom panel of Figure 7A, we indicate the behavioral periods for which we test for the presence of triple-wise spike correlation (the PP and RT to MT; in later analysis we divide the PP into early and late stages). The evidence for model 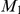, as opposed to 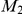, in the behaviorally relevant time periods is obtained by summing the log of the bin-by-bin BF in that period (cf. Eqs. 12 and 13). The BF in the PP is found to be 18.08 bit, which is interpreted as ‘very strong’ evidence for presence of a positive triple-wise spike correlation by the classical guideline [76].

Next, we test the observed value of the BF in the PP (18.08 bit) by comparing it with surrogate BFs. These are computed from null data that contain no triple-wise spike interactions, while keeping the time-varying structure of the pairwise correlations and the individual spike rates the same as those observed in the original spike train data. The construction of the null data follows the same procedure as in the simulation study shown before: We apply a pairwise state-space log-linear model (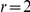) to the spike data and then generate 1000 surrogate samples (each with 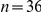 trials) of 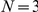 parallel spike sequences using the fitted pairwise state-space log-linear model. Figure 7B (left panel) displays the surrogate distribution along with the observed BF in the PP. The observed positive BF falls out of the 95% confidence interval, suggesting the presence of a positive triple-wise interaction as an underlying model for the spike train data in this period. In the right panels of Figure 7B, we display the results of the same analysis, but using spike train data binned by a larger (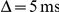, top panel) and a smaller (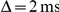, bottom panel) bin-width. We obtained almost the same results as in the analysis with a bin-width of 3 ms as for the analysis with the larger bin-width (5 ms). However, we could not reach the same results for the smaller bin width (2 ms) because the small count of synchronous spike events made it impossible to reject the null hypothesis.

If the higher-order dependency among the three neurons is related to motion preparation, the evidence for the triple-wise spike correlation should be stronger in the late stage of the PP than before. To test this idea, we divided the PP into earlier and later stages of the PP (each with a 750 ms duration, see the bottom of Figure 7A), and investigate whether the three MI neurons exhibit a triple-wise spike correlation in these periods. In addition, we select a duration of 300 ms after the onset of the RS, during which the animal starts to initiate the motor action (reaction and movement time, RT-MT). The upper panels of Figure 7C display the results obtained with bins of width 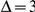 ms. The weights of evidence for the triple-wise spike correlation models are 7.16, 11.2, and 0.98 bit in the early PP, late PP, and RT-MT periods respectively: i.e., ‘very strong’ evidence is found at the late stage of the PP. The null hypothesis is rejected in this late period of the PP whereas the same null hypothesis is not rejected in the earlier period of PP or the period after the RS. In the late PP, the significantly large positive BF indicates the existence of a positive triple-wise spike correlation. The bottom panels of Figure 7C display the same analyses, but with a larger (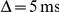) bin-width. We obtain the same results as in the analysis with a bin-width of 3 ms. When we analyze the data using a smaller bin-width (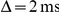), the BFs are not significant in any of the periods (not shown here) because of the small samples of synchronous spikes. In addition, as in the simulation study in the previous subsection, we tested whether the observed synchronous spiking activities are explained merely by simultaneous increases in the pairwise interaction terms of the second order log-linear model. We did not find any evidence for such simultaneous increases in the pairwise interactions from the data for all three periods (bin-width 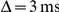) (not shown here). Thus only by the application of the higher-order analysis we were able to detect the task-dependent changes in the joint interactions of all three neurons.

The critical assumptions made in this study are independence and identical sampling across the trials (across-trial stationarity). However, in the spike sequences of neuron 2 (neuron id 3) shown in Figure 6B, we notice an increase in the firing rates across trials. Higher firing rates yield a higher chance rate for synchronous spiking events. Thus, underestimation of the spike rate caused by averaging across the entire trials might induce spurious estimation of higher-order spike interaction. Indeed, synchronous spike events of the three neurons are mostly observed in later trials (e.g., trials 19–36, see blue dots in Figure 6B) when the firing rates were higher than in the earlier trials. Hence, we repeat the same analysis by using only the latter half of the trials (trials 19–36). Figure 7D displays the results. We still observe a significantly positive BF in the late PP. We note that the estimated dynamics of the log-linear parameters using only the latter half of the trials are not changed much from those using all trials, i.e., Figure 6D. In the analysis using the first half of the trials (1–19, not shown), the BF is not significant during the late PP. Kilavik et al. [19] reported that “during practice, the temporal structure of synchrony was shaped, with synchrony becoming stronger and more localized in time during late experimental sessions”. We observe similar effects in our triple-wise spike correlation analysis.

## Discussion

In this study, we introduced a novel method for estimating dynamic spike interactions in multiple parallel spike sequences by means of a state-space analysis (see Methods for details). By applying this method to nonstationary spike train data using the pairwise log-linear model, we can extend the stationary analysis of the spike train data by the Ising/spin-glass model to within-trial nonstationary analysis (Figure 3). In addition, our approach is not limited to a pairwise analysis, but can perform analyses of time-varying higher-order spike interactions (Figure 4). It has been discussed whether higher-order spike correlations are important to characterize neuronal population spiking activities, assuming stationarity in the spike data [54]–[59]. Based on the state-space model optimized by our algorithm, we developed two methods to validate and test its latent spike interaction parameters, in particular the higher-order interaction parameters, which may dynamically change within an experimental trial. In the first method, we selected the proper order for the spike interactions incorporated in the model under the model selection framework using the approximate formula of the AIC for this state-space model (In Methods, ‘Selection of state-space model by information criteria’). This method selects the model that best fits the data overall across the entire observation period. The selected model can then be used to visualize the dynamic spike interactions or for a performance comparison with other statistical models of neuronal spike data. However, more importantly, the detailed structure of the transient higher-order spike interactions needs to be tested locally in time, particularly in conjunction with the behavioral paradigm. To meet this goal, we combined the Bayesian model comparison method (the Bayes factor) with a surrogate method (In Methods, ‘Bayesian model comparison method for detecting spike correlation’). The method allows us to test for the presence of higher-order spike correlations and examine its relations to experimentally relevant events. We demonstrated the utility of the method using neural spike train data simultaneously recorded from primary motor cortex of an awake monkey. The result is consistent with, and further extended the findings in the previous report [8]: We detected an increase in triple-wise spike interaction among three neurons in the motor cortex during the preparatory period in a delayed motor task, which was also tightly locked to the expected signals. Although the analysis was done for a limited number of neurons, smaller than the expected size of an assembly, it demonstrates that the nonstationary analysis of the higher-order activities is useful to reveal cooperative activities of the neurons that are organized in relation to behavioral demand. Of course, further analysis is required to strengthen the findings made above including a meta-analysis of many different sets of multiple neurons recorded under the same conditions.

**Figure 8:**
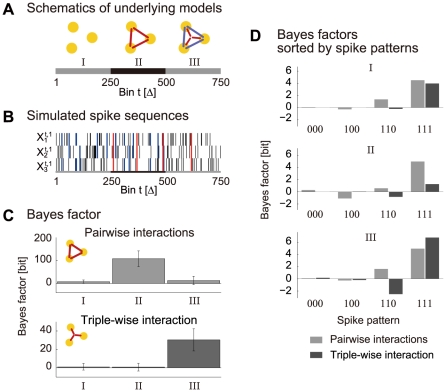
Analysis of stationary spike correlations of 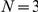 simulated neurons using Bayes factor. (A) Sketch of different time periods and the underlying models used for the generation of parallel spike sequences: (I) Model of independent spiking (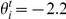, 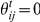, 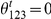 for 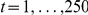 and 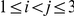); (II) Model of simultaneously positive pairwise interactions, without a triple-wise interaction (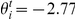, 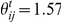, 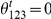 for 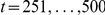); (III) Model of triple-wise interaction, with negative pair interactions (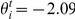, 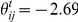, 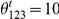 for 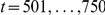). (B) Raster display of three parallel spike sequences, 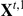, within one example trial. Each spike is colored according to the spike pattern in which it appears: Spikes occurring in triplets are shown in red, spikes within doublets (all types) are marked in blue, and spikes not involved in any synchrony pattern are shown in black. (C) The bar plots demonstrate the Bayes factors (BFs), Eq. 48, for each of the time periods I–III in bit units. The upper panel shows the average BF when testing simultaneously positive pairwise interactions (Test 1), averaged across 200 realizations (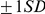). A positive value for the log of the BF supports the model for the presence of simultaneously positive pairwise interactions, 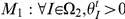, while a negative value supports the absence of such an assembly, 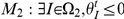. The BFs per sample and time period are computed by applying a pairwise state-space log-linear model (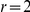) independently to the three periods. We use a state model with 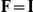. The lower panel shows the BF for the positive triple-wise spike interaction (Test 2), 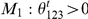, as opposed to a zero or negative triple-wise spike interaction, 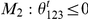, in each of the three periods. The BFs are computed from a full state-space log-linear model (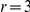). (D) Bar plot of the bin-by-bin BFs (Eq. 52) sorted by the different spike patterns in the three periods (from top to bottom). The contributions to the BFs (shown in C) from the different spike patterns are sorted and displayed using the indicated spike patterns (000, 100, 110 and 111) as representative examples. The gray bars indicate the average BFs of simultaneously positive pairwise interactions, with the average computed for the spike patterns observed in 200 realizations in the respective periods, while the dark gray bars indicate the average BFs for the triple-wise spike correlation.

In this study, we adopted the log-linear model to describe the higher-order correlations among the spiking activities of neurons. There are, however, other definitions for ‘higher-order spike correlation’. An important alternative concept is the definition based on cumulants. Using the cumulants of an observed count distribution from a spike train pooled across neurons, Staude et al. developed an iterative test method that can detect the existence of a high amplitude in the jump size distribution of the assumed compound Poisson point process (CPP) model for the pooled spike train [34], [35]. This method can detect an assembly from a few occurrences of synchronous spike events to which many neurons belong to, typically by using the lower-order cumulants of the observed spike counts. In contrast, the information geometry measure for the higher-order spike correlation used in this study aims to represent the correlated state that cannot be explained by lower-order interactions. Consequently, the information geometry measure extracts the relative strength of the higher-order dependency to the lower-order correlated state. Therefore, the presence of positive higher-order spike correlations does not necessarily indicate that many neurons spike synchronously whenever they spike because such activities can be induced by positive pairwise spike correlations alone [65], [85], [86] (see also Figure 8A and B in the Methods section). In contrast, the cumulant-based correlation method by Staude et al. [34] infers the presence of ‘higher-order correlation’ for such data by determining the presence of high amplitudes in the jump size distribution of the assumed CPP model. Yet another important tool for analyzing higher-order dependency among multiple neurons is the copula function, a standardized cumulative distribution function used to model the dependence structure of multiple random variables (see [87]–[89] for an analysis of neurophysiological data using the copula, including an analysis for higher-order dependency [89]). In summary, it should be remembered that the analysis method used for the higher-order dependency among neuronal spikes inherits its goal from the assumed model for spike generation as well as a parametric measure defined for the ‘higher-order’ spike correlation [34], [62].

Although we face a high-dimensional optimization problem in our settings, we are able to successfully obtain MAP estimates of the underlying parameters because of the simplicity of the formulation of the state-space model: The use of the log-concave exponential family distributions [50], [90] in both the state and observation models guarantees that the MAP estimates can be obtained using a convex optimization program. At each bin, the method numerically solves a nonlinear filter equation to obtain the mode of the posterior state density (the MAP estimates, see Eqs. 28, 29, and 30 in Methods). With only a few (3–8) Newton-Raphson iterations, the solution reaches a plateau (the increments of all the elements of the updated state space vector are smaller than 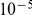). The entire optimization procedure can be performed in a reasonable amount of time: On a contemporary standard laptop computer it takes no longer than 30 seconds to obtain smooth estimates of a full log-linear model for 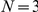 neurons (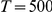 bins, Figure 4), which includes 100 EM iterations. The method is even faster when approximating the posterior mode using the update formula Eq. 30 without any iterations, using the one-step prediction mean as an initial value. This fast approximation method suggested in [69] could even be utilized in a real-time, on-line application of our filter (the filtering method applied to a single trial, 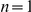, using predetermined hyper-parameters) at the cost of estimation accuracy.

The pairwise analysis can be applied up to about 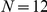 neurons simultaneously to derive time-dependent pair interactions. However, the current version of the algorithm does not scale to a larger number of neurons because the number of spike patterns that need to be considered suffers from a combinatorial explosion. The major difficulty arises from the coordinate transformation from the 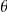-coordinates to the 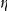-coordinates that appear in the non-linear filter equation (Eq. 31 in Methods). The coordinate transformation is required in this equation to calculate the innovation signal, i.e., the difference between the observed synchrony rates, 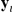, and the expected synchrony rates (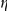-coordinates) based on the model. We numerically derived the exact 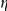-coordinates by marginalizing the 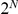 dimensional joint probability mass function computed from the 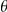-coordinates. Thereby, a full knowledge of the probability mass function is required even if the model considers only the lower-order interactions. Because this is a common problem in the learning of artificial neural networks [46], [47], [91], sampling algorithms such as the Markov chain Monte Carlo method have been developed to approximate the expectation parameters, 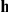, without having to compute the partition function [92]. The inclusion of such methods allows us to analyze the time-varying low order spike interactions from a larger number of parallel spike sequences. Recent progress [93], e.g., in the mean field approach and/or the minimum probability flow learning algorithm for an Ising model, may allow us to further increase the number of neurons that can be treated in this nonstationary pairwise analysis. Nonetheless, the method presented here, which aims at a detailed analysis of the dynamics in higher-order spike interactions, may not easily scale to massively parallel spike sequences that can be analyzed by other methods such as those based on the statistics pooled across neurons. Thus, we consider it to be important to combine the detailed analysis method proposed in this study with other state-of-the-art analysis techniques in practical applications. For example, test methods based on population measures such as the Unitary Event method and cumulant-based inference method [34], [35] allow us to detect the existence of statistically dependent neurons in massively parallel spike sequences. If the null-hypothesis of independence among those neurons is not rejected in these methods, we can exclude those neurons from any further detailed analysis of their dynamics using the methods proposed in this study.

Several critical assumptions made in the current framework need to be addressed. First, it was assumed in constructing the likelihood (Eq. 7) that no spike history effect exists in the generation of a population spike pattern. Second, we assumed the use of identically and independently distributed samples across trials when constructing the likelihood (Eq. 7). The first assumption may appear to be strong constraint given the fact that individual neurons exhibit non-Poisson spiking activities [94]. However, as in the case of the estimation of the firing rate of a single neuron, the pooled spike train across the (independent) trials is assumed to obey a Poisson point process because of the general limit theorem for point processes [30], [31], [95], [96]. This is because most of the spikes in the pooled data come from independent different trials. They are thus nearly statistically independent from each other, even if the individual processes are non-Poisson. Similarly, in our analysis, we used statistics from a pooled binary spike train, assuming independence across trials: The occurrences of joint spikes in the binary data pooled across trials are mostly independent of each other across bins. Because these joint spike occurrences are sparse (i.e., they rarely happen closely to each other in the same trial), it is even more feasible to assume their statistical independence across bins. Third, however, while pooling independent and identical trials (the second assumption) may validate the first assumption of the independence of the samples across bins, that assumption of independently and identically distributed samples across trials has itself been challenged [97], [98] and is known to be violated in some cases, e.g., by drifting attention, ongoing brain activity, adaptation, etc. It is possible that the trial-by-trial jitter/variation in the spike data causes spurious higher-order spike correlation. Thus, as discussed in the section on the application of our methods to real neuronal data, it is important to examine the stationarity of the spike train data across trials. Note that, not only the firing rates, but also the spike synchrony can be shaped on a longer time scale by repeatedly practicing a task [19]. In fact, the current analysis method can be used to examine the long-term evolution of pairwise and higher-order spike interactions across trial sessions by replacing the role of a bin with a trial, assuming within-trial stationarity. It will be a challenge to construct a state-space log-linear model that additionally applies a smoothing method across trials (see [98] for such a method for a point process model).

The present method is left with one free parameter, namely the bin-width 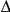. The bin-width determines the permissible temporal precision of synchronous spike events. Very large bin-widths result in binary data that are highly synchronized across sequences, while very small bin-widths result in asynchronous multiple spike sequences. In the latter case, we might overlook the existing dependency between multiple neural spike sequences due to disjunct binning [99] (but see [57], [100] that aim to overcome such a problem by modeling the spike interactions across different consecutive time bins). Within our proposed modeling framework, which focuses on instantaneous higher-order spike correlations, it is important to catch the innate temporal precision of the neuronal population under investigation using the appropriate bin-width. Thus, the choice may be guided by the biophysical properties of the neurons. However, it may be of advantage to derive the bin-width in a data-driven manner. For example, in the context of an encoding problem, the proper bin-width can be chosen based on the goodness-of-fit test for single neuron spiking activities [101], conditional on the spiking activities of the other neurons [40]. For questions about the relation of coincident spiking to stimulus/behavior, the bin-width may be selected based, for example, on the predictive ability of an external signal. For this goal, it is important to search the optimal bin-width using elaborate methods such as those developed in the context of the Unitary Event analysis method [99] (see [25] for a review of related methods).

A substantial number of studies have demonstrated that stimulus and behavioral signals can be decoded simply based on the firing rates of individual neurons. At the same time, it has been discussed whether spike correlations, particularly higher-order spike correlations, are necessary to characterize neuronal population spiking activities [54]–[58] or to encode or decode information related to stimuli [53], [60], [102]. At this point in time, a smaller number of dedicated experiments have supported the conceptual framework of information processing using neuronal assemblies formed by neurons momentarily engaged in coordinated activities, as expressed by temporally precise spike correlations (see [6], [7], [9], [10] for reviews of these experiments). Nevertheless, it is possible that the current perspective on this subject has been partly formed by a lack of proper analysis approaches for simultaneously tracing time-varying individual pairwise spike interactions, and/or their higher-order interactions. Indeed, we demonstrated by the time-resolved higher-order analysis that three cortical neurons coordinated their spiking activities in accordance with behaviorally relevant points in time. Thus our suggested analysis methods are expected to be useful to reveal the dynamics of assembly activities and their neuronal composition, as well as for testing their behavioral relevance. We hope that these methods help shed more light on the cooperative mechanisms of neurons underlying information processing.

## Methods

### Mathematical properties of log-linear model

In this subsection, we review the known mathematical properties of a log-linear model for binary random variables. These properties are used in constructing recursive filtering/smoothing formulas in the next section. Using the multi-index, 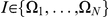 (see the Results subsection ‘Log-linear model of multiple neural spike sequences’), the probability mass function (Eq. 1), 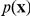, where 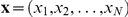 and 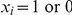 (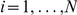), and the expectation parameters (Eq. 2) are compactly written as
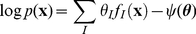
(14)and
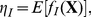
(15)where 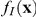 is a feature function, here representing an interaction among the neurons indicated by the multi-index, 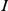 (Eq. 3).

The 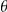
- and 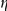-coordinates are dually flat coordinates in the exponential family probability space [44], [50], and the coordinate transformation from one to the other is given by the Legendre transformation [40], [50]. From Eq. 14, the log normalization function, 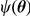, is written as
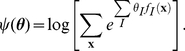
(16)The first derivative of the log normalization function, 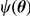, with respect to 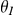 (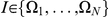), provides the expectation parameter, 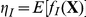:
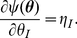
(17)Let 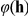 be the negative entropy of the distribution:
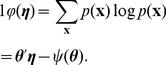
(18)Eqs. 17 and 18 complete the Legendre transformation from 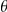-coordinates to 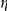-coordinates [44], [50]. The Legendre transformation transfers the functional relationship of 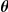 and 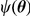 to the equivalent relation in the dual coordinates, 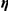 and 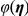. The inverse transformation is given by Eq. 18 and 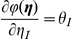.

Using the log normalization function, we can obtain the multivariate cumulants of 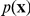 with respect to the random variables, 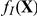. The cumulant generating function of the exponential family distribution is given as 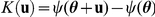. Let us compactly write the partial derivative with respect to 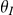 (i.e., 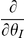) as 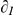. Then, the first order cumulant is given as 
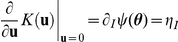
, as shown in Eq. 17. In general, the cumulants of the exponential family distribution are given by the derivatives of the log normalization function. Thus, the second derivative of 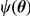 yields the second-order cumulant, 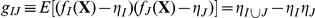 (by the cup, 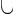, we mean the multi-index representation of an union of the elements of the two multi-indices, e.g., if 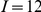 and 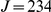, then 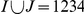):
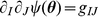
(19)for 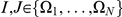. 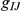 is known as the Fisher metric with respect to the natural parameters. Eqs. 17 and 19 are important relations used in this study to construct a non-linear filtering equation for a dynamic estimate of the natural parameters because we approximate the log-linear model (Eq. 14) with a precision of up to a (log) quadratic function (cf. Eqs. 28 and 29). Similarly, the higher-order derivatives yield higher-order multivariate cumulants. For example, the third-order derivative yields the third order cumulant, 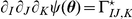, where 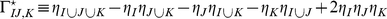.

The pseudo distance between two different distributions, 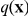 and 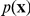 is defined using the Kullback-Leibler (KL) divergence
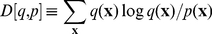
(20)We represent distribution 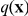 by using 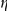-coordinates as 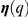, and 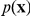 by using 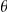-coordinates as 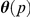. Here, we used 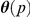 for the 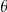-parameters of 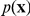 (and 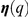 for 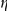-parameters of 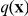) in order to differentiate it from the representation of distribution 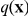 in the 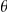-coordinates (and the representation of 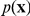 in the 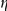-coordinates). Then, the KL-divergence between the two distributions, 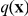 and 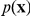, is computed as [44], [50]

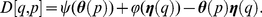
(21)


### Optimized estimation of dynamic spike interactions

We develop a non-linear recursive Bayesian filtering/smoothing algorithm and its optimization method in order to trace dynamically changing spike interactions from parallel spike sequences. To reach this goal, we use the expectation-maximization (EM) algorithm [68], [73], [103], [104], which is known to efficiently combine the construction of the posterior density of a state (the natural parameters) and the optimization of the hyper-parameters. This method maximizes the lower bound of the log marginal likelihood, Eq. 10. Using Jensen's inequality and nominal hyper-parameters, 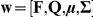, the lower bound of the log marginal likelihood with hyper-parameters 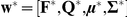 is given by
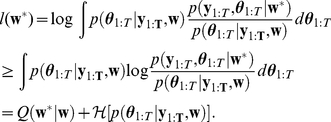
(22)Here, 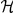 represents a negative entropy. The maximization of the lower bound with respect to 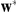 is equivalent to maximizing the expected complete data log-likelihood in Eq. 22, known as the 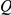-function:
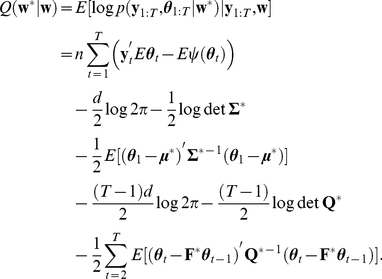
(23)The expectation in the above equation is read as 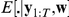. We maximize the 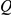-function by alternating the expectation (E) and maximization (M) steps. In the E-step, we obtain the expected values with respect to 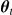 in Eq. 23 using a fixed 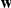. In the M-step, we obtain the hyper-parameter, 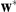, that maximizes Eq. 23. The resulting 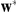 is then used in the next E-step. The details of each step are now given as follows.

#### E-step: Bayesian recursive filter/smoother

The E-step is composed of filtering and smoothing algorithms conducted by forward and backward recursions, respectively. The forward algorithm sequentially constructs a posterior density of the state at time 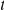 given the spike data up to and including time 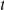, whereas the backward algorithm constructs a posterior density at time 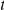 given the entire data. The posterior density allows us to compute the maximum a posteriori (MAP) estimate or Bayes estimator and provides uncertainty for the estimate. In the following, 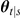 and 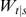 denote the conditional mean, 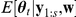, and covariance, 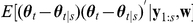. The filter mean and covariance are denoted as 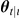 and 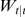, respectively. The mean and covariance of a smooth posterior density are denoted as 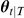 and 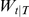, respectively.

We first compute the one-step prediction density, 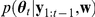. This is the conditional density of the state at time 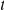 given the observation of parallel spike sequences up to time 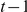. The one-step prediction density is written using the Chapman-Kolmogorov equation as [37], [69], [105]

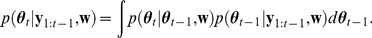
(24)Here the transition probability, 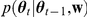, is a multivariate normal distribution with mean 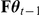 and covariance 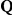, as defined in the state equation, Eq. 8. For an initial prior, 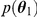, we use a normal distribution with mean 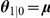 and covariance 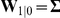. The other distribution, 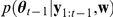, in Eq. 24 is the filter density at time 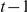. In the next paragraph, the filter density will be obtained by approximating it with a normal distribution whose mean and covariance are denoted as 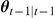 and 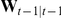. Under this condition, the one-step prediction density (Eq. 24) again becomes a normal distribution whose mean, 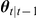, and covariance, 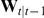, are given as [37], [68]

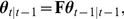
(25)

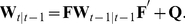
(26)


The filter density, 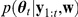, is the conditional distribution of the state given the observation of parallel spike sequences up to time 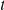. Using the likelihood function and one-step prediction density, the filter density is given by Bayes' theorem as
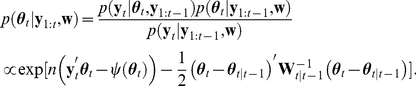
(27)This posterior density is a complicated function with respect to the natural parameter, 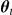. Here, we apply a Gaussian approximation to the posterior density using the Laplace method [37], [104], [106], [107]: The filter mean, 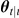, is identified with a mode of the posterior density as 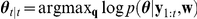, and the filter covariance, 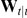, is determined from the Hessian of the log posterior at the mode as 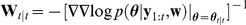. The approximate posterior mode is obtained using the iterative procedure of a gradient ascent method or the Newton-Raphson method using the gradient and Hessian matrix. The gradient and Hessian of the log of the posterior density at 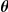 is calculated as
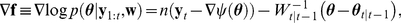
(28)

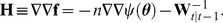
(29)Note that, as in Eqs. 17 and 19, the first derivative of the log normalization function, 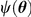, with respect to 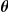 provides the dual coordinates, 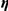: 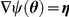. Furthermore, the second derivative yields the Fisher metric: 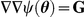. In this study, we adopt the Newton-Raphson method:
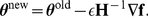
(30)The gradient and Hessian, Eqs.28 and 29, are evaluated using an old value, 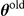. Here, 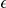 is a learning coefficient that was introduced in the context of ‘natural’ gradient search algorithm [91]. For the small population size analyzed in this study, we used 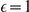. However, it is recommended that a smaller positive value be selected for the analysis of a larger system to avoid numerical instability. Because both the likelihood function and prior density are logarithmically concave functions, the posterior is also log-concave. Thus, this optimization problem is convex, which guarantees a unique solution for the filter estimate [90]. The optimized natural parameter is selected as the filter mean, 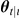. The filter covariance is approximated as 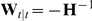 by using 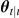. It is also possible to use a simple gradient ascent method to obtain the mode, and then compute the Hessian matrix at the mode.

The above method is equivalent to solving the following nonlinear recursive filter formulas:
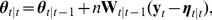
(31)

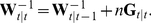
(32)Eq. 31 was obtained from 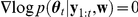. Eqs. 31 and 32 are recursively computed in combination with the one-step prediction equations, Eqs. 25 and 26, for 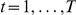. Because 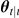 and 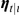 are dual representations of the same probability distribution, Eq. 31 is a nonlinear equation and needs to be solved as suggested above (Eqs. 28, 29, 30). As pointed out for a point process adaptive filter [37], [69], the residual, 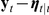, in Eq. 31 acts similarly to an innovation vector of a standard Kalman filter. The same error signal between the observed synchrony rates and expected synchrony rates is utilized in training the Boltzmann machine [46], [47], [91]. The innovation term corrects the one-step prediction mean, 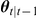, i.e., an expected state by the prior distribution. The degree of the correction is determined by the number of repeated trials, 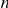, and uncertainty of the predicted state, 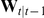. The hyper-parameters of the prior density significantly affect the latter gain (see Eq. 26), and thereby the smoothness of the estimated processes. The filter covariance equation, Eq. 32, describes the reduction of the prediction uncertainty, 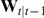, by observing the parallel spike sequences at time 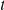, with the amount determined by the number of repeated trials, 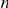, and the Fisher information, 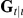. Please see Figure 1 for a geometric view of the recursive Bayesian filter.

Finally, we compute the smooth posterior density using the backward recursive algorithm [68], [105], [107],
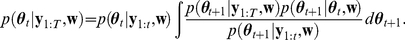
(33)Because the density functions in the recursive formula were approximated as a normal distribution, we follow the fixed-interval smoothing algorithm [37], [68], [105] established for a Gaussian state and observation equation. Starting from 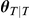 and 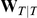, which are obtained from the filtering algorithm, we obtain the smoothed mean and covariance,
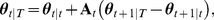
(34)

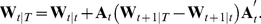
(35)with
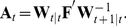
(36)for 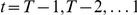. The lag-one covariance smoother, 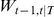, which appears in the 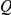-function, is obtained using the method of De Jong and Mackinnon [108]:
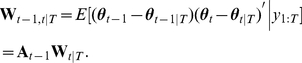
(37)


#### M-step: Optimization of hyper-parameters

At the M-step, we optimize hyper-parameter 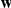 given the posterior density under the principle of maximizing the 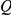-function. From 
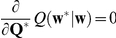
, the update rule of the covariance matrix, 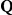, is obtained as
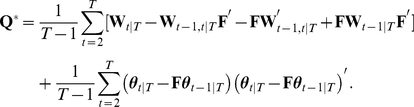
(38)Here, 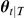, 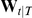, and 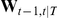 are the smoother mean and covariance, and the lag-one covariance matrix given by Eqs. 34, 35, and 37, respectively. Similarly, from 
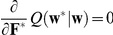
, the auto-regressive parameter, 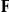, is updated according to
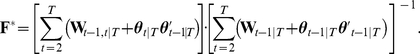
(39)The mean of the initial distribution is updated with 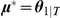 from 
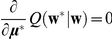
. The covariance matrix, 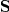, is not updated; instead, we use a fixed matrix, 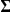. Alternatively, the covariance matrix of the initial distribution can be updated according to 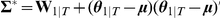 from 
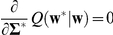
, while the mean, 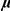, is fixed. Both methods work well with appropriate choices for the fixed parameters. In this study, we updated the mean vector, 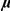, of the initial normal distribution, and used a fixed diagonal matrix for its covariance matrix, 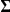. It was also suggested to use a stationary mean and covariance of an unconstrained state process (Eq. 8) as parameters of the initial prior distribution [68], [109]. The equilibrium condition from Eq. 8 yields 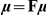 and 
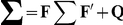
. The solutions are obtained as 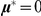 and 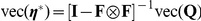 (p. 121 and p. 426 in [109], p. 112 in [110]). In the latter, 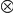 denotes the Kronecker product (tensor product) and the 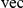 operator creates a single column vector from a matrix by stacking its column vectors. However, the stationary condition is not always satisfied. As we found that the fitted 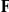 sometimes violates the stationary assumption, we did not adopt this approach in the current study.

### Selection of state-space model by information criteria

The method developed in the previous subsection is applicable to a full log-linear model, as well as a model that considers an arbitrary order of interactions. In order to select the most predictive model among the hierarchical models in accordance with the observed spike data, we select the state-space model that minimizes the Akaike information criterion (AIC) for a model with latent variables [73], [105]:
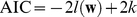
(40)Here, 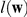 is the log marginal likelihood (Eq. 10) and 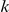 is the number of free hyper-parameters in the prior distribution. For the 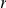
th order model, the number of natural parameters is given by 
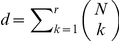
. The number of free parameters in the prior distribution is computed as 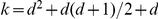, where each term corresponds to the number of free parameters in 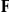, 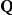, and 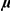. Note that the AIC applied to the state-space model is sometimes referred to as the Akaike Bayesian information criterion (ABIC) [73]. In the following, we derive the approximation method to evaluate the AIC for the state-space log-linear model.

The log marginal likelihood, Eq. 10, can be written as
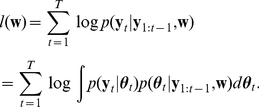
(41)We make a log quadratic approximation to evaluate the integral. To accomplish this, we denote
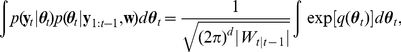
(42)with
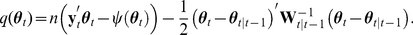
(43)The Laplace approximation of the integral in Eq. 42 is given as [80]

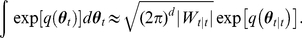
(44)By applying Eqs. 42, 43 and 44 to Eq. 41, the log marginal likelihood is approximated as
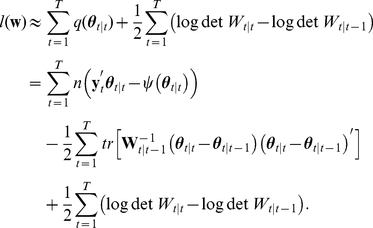
(45)We confirmed that the log-quadratic approximation provided a better estimate of the marginal likelihood than the first order approximation used in [77] by comparing them with a Monte Carlo approximation of the integral in Eq. 42. We select the state-space log-linear model that minimizes the AIC (Eq. 40), where the log marginal likelihood is approximated using Eq. 45.

For comparison with the AIC, we compute two other information criteria that employ different forms of the penalization term. The Bayesian information criterion [79], [80] (also known as Schwartz's criterion) are obtained by replacing the penalization term of Eq. 40, 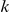, with 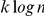:
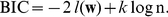
(46)Shimodaira's predictive divergence for indirect observation models (PDIO) [81] is given as
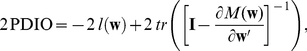
(47)Here, we redefine 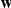 as a one-dimensional vector of free hyper-parameters, while 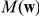 denotes the one-step operator of EM iteration. To obtain the Jacobian matrix for the EM operator, we follow the algorithm described in Meng and Rubin [111]. In this method, the Jacobian matrix was approximated using a numerical differentiation of the EM operator. By perturbing one hyper-parameter and then computing a one-step EM iteration, numerical differentiations of the hyper-parameters with respect to the perturbed hyper-parameter were obtained. An entire Jacobian matrix was approximated by repeating the process while changing the hyper-parameter to be perturbed.

### Bayesian model comparison method for detecting spike correlation

In this subsection, we formulate a method for detecting the hidden structure of spike interaction by means of a Bayesian model comparison based on the Bayes factor (BF) [74]–[76]. The BF is a ratio of likelihoods for the observed data, based on two different assumptions about the hidden states (model 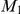 and 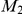). Here we reiterate the definition of the BF for model 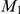 as opposed to model 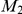 used in this paper (cf. Eq. 12):
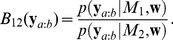
(48)The BF becomes larger than 1 if the data, 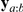 in a time period 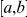, supports model 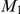 as opposed to model 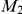, and becomes smaller than 1 if the data supports model 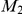 as opposed to model 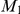. The BF can be computed from the one-step prediction and filter density obtained in the method developed in the preceding subsection. From Eq. 48, the BF can be rewritten as
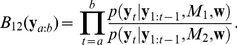
(49)Let us define the bin-by-bin BF for the spike data at time 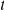 as
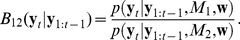
(50)Using Bayes' theorem, we obtain [74]–[76]

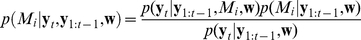
(51)for 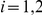. Using Eq. 51, we can rewrite the bin-by-bin BF as
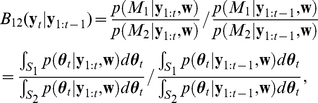
(52)where 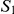 and 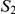 denote spaces that the natural parameters occupy, supported by models 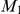 and 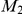, respectively. Here, 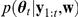 and 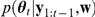 are the filter density and one-step prediction density, respectively. By sequentially computing Eq. 52, we can obtain the BF with respect to the sub-interval 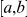 as 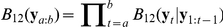.

A test with the following models in the sub-interval 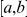 allows us to detect a momentarily active cell assembly of more than two neurons by the presence of simultaneously positive 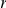
th-order spike interactions. In the 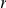
th-order log-linear model of 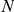 neurons, the natural parameters, 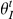 (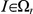), represent the 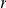 th-order spike interactions among neurons denoted in index 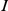. We examine whether a subset of 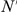 neurons among the total 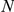 neurons simultaneously exhibit positive 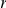 th-order interactions. Let 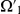 be the subset of 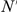 neurons from 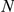 neurons, e.g., 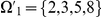 from 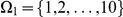 if 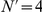 and 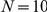. Let 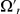 be an 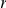-subset from 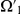, e.g. 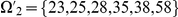 if 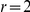. Then, the model where the subset neurons simultaneously exhibit positive 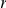
th-order interactions (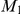) and its complementary hypothesis (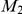) are represented as
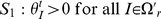
(53)

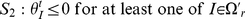
(54)for 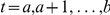. The remaining parameters are real: 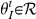 (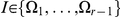 and 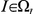. excluding 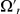). These parameters are integrated out in Eq. 52. The above definition of the assembly is a clique [12], a subset in which each neuron is connected to every other neuron through the positive 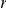
th-order interactions. Depending on the assembly structure one wishes to uncover, other models can be tested such as one in which neurons bounded in non-exclusive manner.

#### Analysis of Bayesian model comparison method using simulated stationary spike sequences

In this subsection, we analyze the Bayesian model comparison method by using simulated stationary spike sequences. Figure 8 illustrates an analysis where the BF is applied to stationary spike sequences of 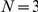 simulated neurons. To demonstrate the kind of correlation scenarios that is distinguished by the BF, we generate stationary spike sequences using log-linear models of different spike correlation structures in three distinct time periods (I–III, Figure 8A). These are: (I) an independent model (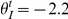 for 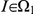 and 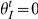 for 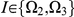 at 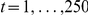), (II) a model containing simultaneously positive pairwise interaction terms, without a triple-wise interaction (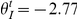 for 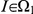, 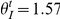 for 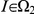, and 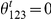 at 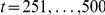), and (III) a model containing a positive triple-wise interaction, with negative pairwise interactions (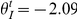 for 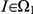, 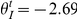 for 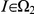, 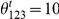 at 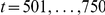). A sample of the spike sequences from a single trial (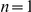) is shown in Figure 8B. In all of the models, the first order log-linear parameters, 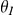 (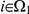), are adjusted such that the spike rates of the individual neurons are 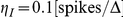 (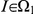). Therefore, the time segments cannot be distinguished on the basis of the firing rates. In addition, the model in period III is designed such that the projection to 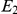 yields zero pairwise interactions, i.e., the projection yields an independent model. In this model, the second-order joint synchrony rates, 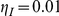 (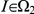), are at the chance level expected from the individual spike rates, 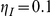 (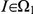). However, the model expresses excess joint spike synchrony between all three neurons, 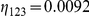, which is larger than the expected rate (0.001). The presence of a positive triple-wise correlation, combined with the absence of marginal pairwise correlations, leads to a very sparse number of excess synchronous spike triplets, as illustrated by the dot displays in period III in Figure 8B. In contrast, a period containing only positive pairwise correlations without a triple-wise interaction (see Figure 8B, period II) contains a relatively large number of triplets, which are, however, the mere expression of excess pairwise correlations.

We calculate the BFs to test for the presence of the two different correlation structures, i.e., the same tests discussed in the Results section: Test 1 checks for the presence of simultaneously positive pairwise interactions and Test 2 checks for the presence of a triple-wise spike correlation. In one test (Test 1) we ask whether the three neurons simultaneously exhibit positive pairwise interactions by applying a model that contains up to pairwise interactions (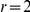) to each of the three time periods. In that case, the BF (Eq. 48) is computed using a model which assumes that all of the pairwise terms are positive: 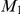, specified as 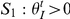 for all 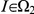, as opposed to a model where at least one of the pairwise interactions is not positive: 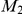, specified as 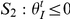 for at least one 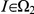. Time (bin) index, 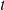, expresses that the BF is computed under the same models for all time steps 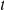 in the respective time period. In both models, we do not make specific assumptions for the first order log-linear parameters; thus, they are allowed to be real numbers. In the other test (Test 2), we ask whether the neurons exhibit excess triple-wise synchrony by applying a full model (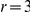) to each of the periods. Here, the BF is computed with the opposing models: 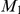, specified as 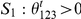, and 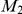, specified as 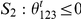. The lower-order log-linear parameters have again real values.

Figure 8C displays the average BFs (in units of bits) computed from 200 samples (each sample contains 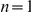 trial) within each of the periods I–III. In addition, we show the standard deviations of the BFs (shown as 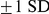) derived from the BFs of the 200 realizations. The upper panel shows the result of Test 1, expressing the weight of evidence for the presence of excess synchrony realized by simultaneously positive pairwise interactions. We find substantial evidence for the simultaneously positive pairwise interactions in period II, but not in the other two periods. The lower panel displays the evidence for the presence of a positive triple-wise correlation as evaluated by Test 2. The existing positive triple-wise interaction present in period III is well detected by the large BF, and the very low BFs in period I and II correctly detect the absence of triple-wise correlation.

In Figure 8D, we examine how much each individual spike pattern contributes to the calculation of the BFs discussed above. The weight of evidence (log of the BF) in an observation period 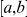 is obtained by summing the pieces of evidence computed locally at time 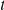 using Eq. 52, as 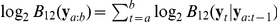. Here we elucidate the contributions of each individual spike pattern to the BF by sorting the bin-by-bin evidence, 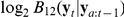, with each spike pattern. Figure 8D displays the average BFs for each spike pattern in the three periods in bit unit. The degree of the contribution by a specific spike pattern to the evidence for the tested spike interaction model varies depending on the context in which the spike pattern is observed. For example, the magnitudes of the BFs for a triple-wise correlation (Test 2) found by observing a spike triplet (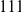) vary greatly across the three periods (see dark gray bars at 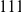 in periods I, II, and III). In period III, where pair-synchronous spikes are rarely observed because of the existence of negative pairwise interaction terms, the observation of triplet spikes provides substantial evidence of a triple-wise correlation. In contrast, in period II, where pair-synchronous spikes frequently occur because of the existence of positive pairwise interactions, the chance triplet spikes do not provide substantial evidence of a triple-wise correlation. These results come from the fact that an unexpected synchronous spike pattern that significantly alters and updates the filter density from its one-step prediction density gives rise to a large absolute BF value (Eq. 52). By the same logic, the BFs for the 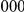 and 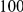 patterns (and 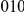 and 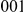, not shown) are small because they do not substantially change the posterior densities. However, they should not be neglected because these patterns are abundant as a result of the low firing rates. For example, in period I in Figure 8C, the accumulation of the small weight of evidence from the abundant spike patterns (such as 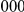 and 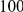) offsets the large weight of evidence induced by a few chance coincidences such as triplet spikes (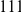), thus producing virtually zero weight of evidence for this period.
